# Lettuce fortification through vitamin B_12_ ‐producing bacteria – proof of concept study

**DOI:** 10.1002/jsfa.14095

**Published:** 2025-01-20

**Authors:** Sara Pipponzi, Stefanie Primisser, Livio Antonielli, Emilio Stefani, Stephane Compant, Angela Sessitsch, Tanja Kostic

**Affiliations:** ^1^ Department of Life Sciences University of Modena and Reggio Emilia Reggio Emilia Italy; ^2^ Center for Health & Bioresources, Bioresources Unit AIT Austrian Institute of Technology Tulln Austria; ^3^ Present address: Institute for Plant Health Laimburg Research Centre Laimburg 6 Auer (Ora) 39040 South Tyrol Italy

**Keywords:** cobalamin, vitamin B_12_ metabolic pathway, biofortification, beneficial endophytes, *Methylobacterium*

## Abstract

**BACKGROUND:**

Vitamin B_12_ (cobalamin) can be produced *de novo* only by certain bacteria and archaea. It plays a crucial role in the health of animals and humans, which obtain it only through diet, mainly from animal products. This study aimed to identify endophytic bacterial strains capable of synthesizing vitamin B_12_ and enriching edible plants with it as a potential solution for vitamin B_12_ deficiency in vegetarians, vegans, and people with poor diets.

**RESULTS:**

An *in silico* genome analysis was performed on 66 bacterial genomes, including the reference strain *Pseudomonas denitrificans* ATCC 13867, a known vitamin B_12_ producer. The genomes were analyzed using the Rapid Annotations using Subsystems Technology (RAST) server and the MetaCyc database to verify the presence and completeness of the vitamin B_12_ metabolic pathway. The ability of the strains to produce vitamin B_12_ was confirmed with a high‐performance liquid chromatography with diode‐array detection (HPLC‐DAD) analysis of pure culture extracts. Eleven strains produced detectable amounts of vitamin B_12_ under tested conditions. The best performing candidates were further tested for their efficacy in producing vitamin B_12_ in lettuce grown under sterile conditions on Murashige and Skoog (MS) medium with or without CoCl_2_ supplementation. *Methylobacterium* sp. strain P1‐11 produced detectable amounts of vitamin B_12_ *in planta*: 1.654 and 2.559 μg per g of dry weight without and with CoCl_2_ supplementation, respectively.

**CONCLUSION:**

This is the first time a bacterial endophyte was used to produce vitamin B_12_ *in planta*, suggesting that bacterial endophytes could be utilized to enhance the nutraceutical values of plant‐based foods. © 2025 The Author(s). *Journal of the Science of Food and Agriculture* published by John Wiley & Sons Ltd on behalf of Society of Chemical Industry.

## INTRODUCTION

Vitamin B_12_, or cobalamin (Cbl), exists in different analogue forms belonging to a family of complex cofactors^1^ (Supporting Information, Fig. S1).

Vitamin B_12_ is essential to mammals because it is a cofactor for two enzymes: methionine synthase, crucial for the synthesis of purines and pyrimidines,^2^ and methylmalonyl‐CoA mutase (MCM), which is involved in the degradation of some amino acids, odd‐chain fatty acids, and cholesterol.^3^ Vitamin B_12_ is therefore required for the development, myelination, and normal functioning of the central nervous system, normal red blood cell formation, and methyl group translocation in DNA synthesis.^4^, ^5^ The Recommended Dietary Allowance (RDA) for adults is 2.4 μg.^6^

Although vitamin B_12_ deficiency is rare in healthy humans,^7^ it may develop due to several causes, such as pernicious anemia^8^ and other gastrointestinal problems, or a strict vegetarian diet.^9^, ^10^ Vitamin B_12_ deficiency can lead to megaloblastic anemia (abnormal blood cell growth), and prolonged deficiency can lead to nerve degeneration and irreversible neurological damage.^9^

**Figure 1:**
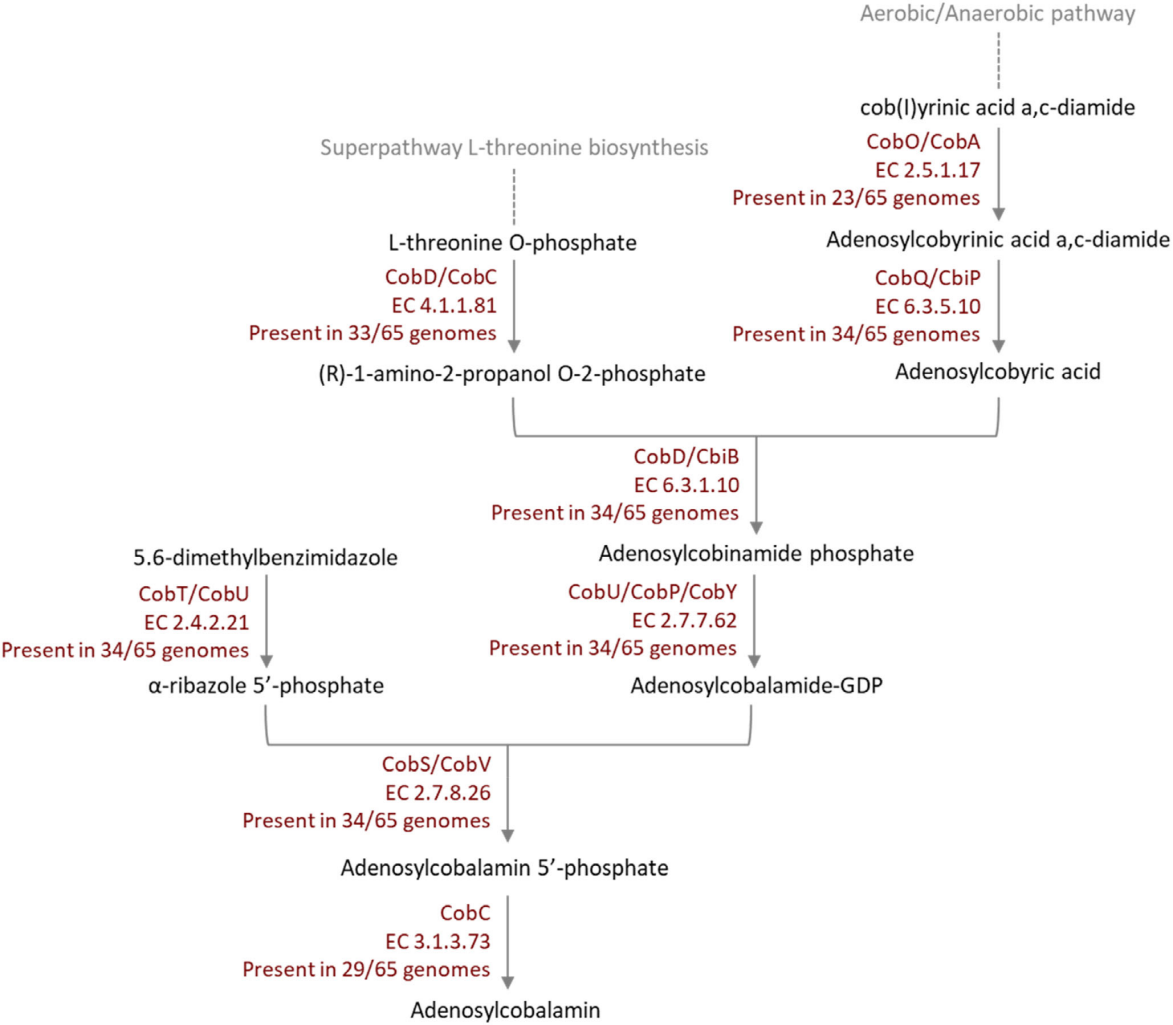
Segment of vitamin B_12_ metabolic pathway, from cob(II)yrinic acid a,c‐diamide to adenosylcobalamin, including the last eight enzymes shared between the aerobic and anaerobic pathways. The number of carrier strains (according to RAST analysis) is indicated for each gene.

Some prokaryotes can produce vitamin B_12_ *de novo* by aerobic or anaerobic metabolic pathways, while some prokaryotes and eukaryotes can take up extracellular cobinamide and convert it to adenosylcobalamin.^11^, ^12^, ^13^, ^14^ The genes/enzymes of the prokaryotic aerobic and anaerobic pathways are defined as *cob*/Cob and *cbi*/Cbi, respectively. Many enzymes in the two pathways are homologues or orthologues, but some are pathway specific. The two metabolic pathways are distinguished based on the timing of cobalt insertion and the oxygen requirement;^15^ however, in the final stage, the two pathways combine again and continue to be fulfilled by structurally similar enzymes^16^ (Fig. 1).

Vitamin B_12_‐synthesizing microorganisms are the source of B_12_ compounds in food. For example, ruminants acquire this essential nutrient through a symbiotic relationship with the microflora inside their stomachs. Consequently, ruminants' meat, milk and, most of all, liver are good sources of vitamin B_12_ for humans.^17^ In aquatic environments, phytoplankton acquires vitamin B_12_ through a symbiotic relationship with bacteria. Then, it becomes food for fish and bivalves, which are also a rich source of vitamin B_12_ for humans.^18^, ^19^ Edible mushrooms like black trumpet (*Craterellus cornucopioides*) and golden chanterelle (*Cantharellus cibarius*) contain considerable amounts of vitamin B_12_: 0.0109–0.0265 μg g^−1^ of dry weight, despite not being able to synthesize it.^20^, ^21^ The vitamin B_12_ in mushrooms is likely to derive from bacteria, as some suggested from a recent study.^22^

Plants neither synthesize nor require vitamin B_12_;^23^ for this reason, plant‐based food is not a source of vitamin B_12_.^24^, ^25^ However, due to the symbiosis between plants and certain vitamin B_12_‐ synthesizing bacteria, it can be found in some plants.^26^ One of these bacteria is nitrogen‐fixing actinobacterium *Frankia alani*, which forms endophytic nodules in woody trees and shrubs.^27^ Actinorhizal plants, such as sea buckthorn (*Hippophae rhamnoides*), *couch grass* (*Elymus repens*), elecampane (*Inula helenium*) or black mustard (*Brassica nigra*), can contain considerable amounts of vitamin B_12_. For example, vitamin B_12_ concentration in sea buckthorn can reach up to 0.37 μg g^−1^ dry weight.^26^

Plant growth‐promoting bacteria (PGPBs) are exploited in agriculture for their ability to improve the quality and growth of their host plants. Beneficial endophytes can stimulate plant growth in multiple ways, either through direct contribution or indirect support. Direct contributions include the provision of nutrients (e.g., N_2_‐fixation) and support in their uptake (e.g., synthesis of enzymes, peptides or siderophores) or the production of phytohormones.^28^, ^29^, ^30^, ^31^, ^32^, ^33^ Endophytes can also indirectly enhance the tolerance against abiotic stresses^29^ and the antagonism to pathogenic organisms by activating mechanisms such as competition for nutrients, production of antibiotics, siderophores, or hydrolytic enzymes.^34^

Beneficial endophytes can also play a role in biofortification strategies that aim to enhance the bioavailable concentration of nutrients and micronutrients in edible portions of food crops through agronomic interventions or genetic selections.^35^, ^36^ Beneficial endophytes can help plants in the acquisition of mineral elements, or they can improve the biosynthesis of secondary metabolites. Biofortification through microorganisms is important because it is an effective and environmentally friendly approach to overcoming nutrient deficiency by growing food crops, especially staple food crops such as cereals, with higher levels of bioavailable nutrients and minerals.^37^

Numerous cases of biofortification targeting mineral elements most commonly lacking in human diets can be found in the literature.^38^, ^39^, ^40^ However, minerals are not the only nutrients that can be targeted through biofortification. In plant‐endophyte interactions, significant changes in the secondary metabolism of the symbionts frequently occur. For example, Khanna *et al*.^41^ observed that *Pseudomonas aeruginosa* and *Burkholderia gladioli* elevated the levels of phenolic compounds, osmolytes (carbohydrates, reducing sugars, trehalose, glycine betaine and proline) and low molecular weight organic acids (fumaric acid, malic acid, succinic acid, and citric acid) in *Solanum lycopersicum* seedlings subjected to cadmium (Cd) stress.

Liu *et al*.^42^ identified 14 bacterial endophytes capable of increasing the accumulation of five Amaryllidaceae alkaloids (narciclasine, lycorine, galanthamine, lycoramine, and tazettine) in leaves, bulbs or roots of *Lycoris radiata* plants. The endophytic bacteria *Luteibacter* spp., isolated from the tea plant, can produce theanine, playing an important role in tea quality.^43^ Furthermore, the vitamin content of plants can also increase through the presence and activity of endophytes, as evidenced by the almost doubled amount of vitamin C found in strawberry fruits produced by plants inoculated with *Phyllobacterium* sp. PEPV15 in comparison with the fruits of uninoculated plants.^44^

The aim of this study was to identify, through genome and biochemical analysis, endophytic bacterial strains able to synthesize vitamin B_12_ *de novo* and attest whether these strains can be exploited to enrich edible plants with this essential nutrient.

## MATERIALS AND METHODS

### Genome sequencing and assembly

A subset of 65 endophytic strains from the bioresources strain collection of the Austrian Institute of Technology (AIT), for which whole genome sequences were available, was selected for this proof‐of‐concept study (Supporting Information, Table S1).

The genome sequencing of selected strains was carried out using the Illumina NextSeq 500/550 Mid Output v2 Kit (Illumina Inc., USA) on the Illumina NextSeq 500 system. The draft genomes were assembled using Evogene Clustering and Assembly Toolbox (EvoCAT), a component of Evogene's MicroBoost AI platform (https://evogene.com/micro-boost/).

The quality of the assembled genomic contigs was evaluated with the Quality Assessment Tool for Genome Assemblies (QUAST)^45^ v5.2. Genome completeness and contamination were assessed using CheckM^46^ v1.2.2. Accurate taxonomic classification was assigned with GTDB‐Tk^47^ v2.3.2, with the R214 GTDB^48^ release serving as the reference database.

### Genome annotation and feature detection

The presence of genes involved in the vitamin B_12_ biosynthetic pathway was investigated using two strategies: first, a Basic Local Alignment Search Tool (BLAST) search of the genomic contigs was performed, aligning them to the reference set of vitamin B_12_ genes;^16^ second, genome annotation was conducted, followed by an inspection of the annotated features. Specifically, the assembled contigs were aligned locally using BLAST+^49^ v2.14.0 against a database of genes associated with vitamin B_12_ production. Hits with at least 70% coverage of the gene sequence length and 80% nucleotide identity^50^ were considered a match.

For the gene search following genome annotation, two complementary methods were employed: Bakta^51^ v1.8.2 (full database v5.0) and the Rapid Annotation using Subsystems Technology (RAST)^52^ annotation server. The genome of *Pseudomonas denitrificans* ATCC 13867 (GCA_000349845.1), a vitamin B_12_ producer used for industrial production,^53^ was also included in the analysis.

The MetaCyc^54^ Metabolic Pathway Database and the information on the vitamin B_12_ metabolic pathway^16^ were used to compare the genes detected by RAST in the different genomes with the enzymes involved in the vitamin B_12_ metabolic pathway. Considering that the aerobic and the anaerobic metabolic pathway for vitamin B_12_ synthesis have the last eight enzymes in common, from the cob(I)yrinic acid a,c‐diamide to the adenosylcobalamin,^16^ the presence of shared genes was investigated. Strains were then selected for further analysis based on the presence and completeness of these genes.

### Bacterial strains cultivation

Putative vitamin B_12_ producers from the Bioresources strain collection and a reference strain, *Pseudomonas denitrificans* ATCC 13867, were used in this study (Supporting Information, Table S1). Cryopreserved bacterial stocks were transferred to 10% Tryptic Soy Agar (TSA, Merck) medium diluted and incubated at 27 °C for 48 h. Afterwards, a single colony of each strain was sub‐cultured in Luria‐Bertani Broth (LB Broth, Merck) supplemented with 0.018 g L^−1^ cobalt chloride (CoCl_2_) to provide cobalt, which is a constitutive element of vitamin B_12_ synthesis.^16^ The pH was adjusted to 6.5 before sterilization (121 °C, 15 min). The bacterial cultures were incubated in the dark for 48 h at 27 °C and at 180 rpm.

### Bacterial vitamin B_12_ extraction and analysis

Vitamin B_12_ present in bacterial cells was extracted as described by Chamlagain *et al*.^55^ Briefly, for each bacterial strain, 1 g of bacterial cell pellet was resuspended in 10 mL of extraction buffer (8.3 mM sodium hydroxide, 20.7 mM acetic acid, pH 4.5). Subsequently, 100 μL of 10 g L ^−1^ potassium cyanide (KCN) solution was added to convert all vitamin B_12_ analogues to the more stable cyanocobalamin. The suspension was mixed using Vortex for 30 s and extracted in a boiling water bath for 30 min, then, the sample was cooled in an ice bath for 10 min and centrifuged at 6000 *g* for 10 min. The supernatant was collected in a fresh tube, and the residual pellet was mixed using Vortex once again with a 5 mL extraction buffer (pH 6.2, adjusted from the pH 4.5 extraction buffer with 30 g L ^−1^ sodium hydroxide) and centrifuged using the same conditions. The supernatants were combined and filtered through Whatman filter paper (Grade 1: 11 μm, VWR). The volume was adjusted to 20 mL with pH 6.2 extraction buffer and filtered again with a syringe filter (RC 0.45 μm, Macherey‐Nagel). The entire extraction process was performed under subdued light conditions to protect the vitamin B_12_ from light degradation.

The analysis of vitamin B_12_ content in the bacterial cells extract was performed with a high‐performance liquid chromatography with diode‐array detection (HPLC‐DAD) (Agilent 1100 series, Agilent Technologies Inc., Santa Clara, California, U.S) consisting of a solvent degasser G1379A, a quaternary pump G1311A, an autosampler G1313A, and a thermostated column compartment G1316A. A Waters 2996 (Waters Corporation, Milford, Massachusetts, U.S) photodiode‐array detector (DAD) was used for detection at 361 nm and full spectra were recorded in the range 330–390 nm with detection every 2 nm. Chromatographic separation was achieved using an Agilent ZORBAX Eclipse Plus C‐18 analytical column (5 μm, 4.6 mm × 150 mm i.d.). Equipment control, data acquisition and integration were performed with Agilent Chem Station HPLC software.

The mobile phase consisted of a mixture of water acidified with 0.25 mL L^−1^ trifluoroacetic acid (pH 2.6) and acetonitrile. The initial setting was 100% water for 0.21 min, which was linearly decreased to 85% water over 2.59 min and then decreased further to 75% over the next 2.4 min. After that, the percentage of water was increased linearly to 90% over 0.24 min and then again to 100% in the next 1.36 min. These conditions were maintained for 4.2 min. The total runtime was 11 min. The flow rate was set to 1 mL min^−1^, the injection volume was 50 μL, and each sample was injected twice. The experiment was carried out at 30 °C.

Vitamin B_12_ in cells extracts was quantified with an external calibration curve obtained by injecting a set of cyanocobalamin (Merck, Darmstadt, Germany) standards eluted pH 6.2 vitamin B_12_ extraction buffer with concentrations of 0, 0.1, 0.2, 0.3, 0.5, 0.75, 1, 1.5 ng μL^−1^. The vitamin B_12_ content in each sample was quantified with the calibration curve after measuring the area integrated from the peaks with a retention time (RT) corresponding to the RT of the vitamin B_12_.

### Plant trial settings

The three best *in vitro* vitamin B_12_ producers, possessing all eight metabolic pathway genes considered (*Pseudomonas* sp. 1489, *Methylobacterium* sp. P1‐11 and *Aneurinibacillus migulanus* C8BA17), and the reference strain *Pseudomonas denitrificans* ATCC 13867 were selected to evaluate their efficacy in producing vitamin B_12_ in lettuce plants.

The strains were grown in the dark for 48 h at 27 °C and 180 rpm in LB Broth enriched with 0.018 g L^−1^ of CoCl_2_, and then the cell suspensions were centrifuged and resuspended in 9 g L^−1^ sodium chloride (NaCl) to obtain an OD_600_ of 1.

The seeds of the lettuce cultivar ‘Chiara’ (ISI Sementi S.p.a., Fidenza, Italy) were surface sterilized by stirring for 2 min in 700 mL L^−1^ ethanol, for 5 min in 50 mL L^−1^ hypochlorite and then five times in sterile MilliQ water for 1 min. The seeds were left to dry on filter paper under sterile conditions for 30 min and subsequently soaked separately for 2 h in the bacterial cell suspensions or 9 g L^−1^ NaCl solution for the negative control.

After incubation, the seeds were sowed in 80 mL glass culture tubes previously filled with 15 mL of Murashige and Skoog (MS) medium (10 g sucrose, 8 g Duchefa Daishin Agar, 1 L MilliQ water, pH 5.8) or MS medium supplemented with 0.018 g L^−1^ of CoCl_2_. The tubes were placed in Plant Growth Chamber SE‐41E2T5 (Percival Scientific Inc, Perry, Florida, U.S) with 14/10 h light/dark and 24 and 20 °C, respectively. Light intensity was 650 μmol m^−2^ s^−1,^ and the light spectrum was 360 nm–780 nm. For each treatment, two replicate sets of 18 plants were prepared. After 25 days, the epiphytic part of 10 plants for each replicate was weighted as a pool and immediately frozen in liquid nitrogen, then freeze‐dried with lyophilizer Alpha 2–4 LSCplus (Christ, Osterode am Harz, Germany). After 5 days, the pools of plants were weighted again and milled into a fine powder with a Mixer Mill MM 400 (Retsch, Korneuburg, Austria). The remaining eight plants were used for bacterial isolation in order to confirm the bacterial colonization of plants. The fresh and dry weights of the epigean parts of lettuce plants were measured as a pool for each replicate and statistically analyzed separately according to the growth medium used (either MS medium or MS medium supplemented with CoCl₂), obtaining four independent groups of values: fresh weights of plants grown on MS medium, fresh weights of plants grown on MS medium supplemented with cobalt chloride, dry weights of plants grown on MS medium, and dry weights of plants grown on MS medium supplemented with cobalt chloride. Within each group, a one‐way analysis of variance (ANOVA) was performed on two replicates using RStudio version 4.1.1, followed by two post‐hoc tests to determine significant differences: Dunnett's test to compare each treatment with the control and Tukey's honest significant difference (HSD) test to compare all treatments with each other.

### Genome annotation

The potential plant growth‐promoting traits (PGPT) of the bacterial strain that showed a positive effect on lettuce growth (*Methylobacterium* sp. P1‐11) were investigated computationally. The genomic protein sequences obtained with Bakta^51^ from the genome of *Methylobacterium* sp. P1‐11 were queried throughout the PLaBAse (PLant‐associated BActeria)^55^ database, applying the PGPT‐Pred tool, which uses BlastP+HMMER approach against the PGPT Ontology.

### Plant vitamin B_12_ extraction and analysis

Vitamin B_12_ was extracted from plants as described by Chamlagain *et al*.^56^ with some modifications. Briefly, for each replicate, 0.1 g of freeze‐dried and milled plant material was resuspended in 10 mL of extraction buffer (8.3 mM sodium hydroxide, 20.7 mM acetic acid, pH 4.5) supplemented with 10 g L^−1^ pepsin and 5 g L^−1^ diastase. Subsequently, 100 μL of 10 g L^−1^ KCN was added. The suspension was sonicated (Sonorex Super RK 255 H, Bandelin, Berlin, Germany) for 15 min at room temperature, shaken at 37 °C and 200 rpm for 2 h (Digital Orbital Shaker, Heathrow Scientific, Veron Hills, Illinois, U.S) and extracted in a boiling water bath for 30 min. It was subsequently cooled in an ice bath for 10 min and centrifuged at 6000 *g* for 10 min. The supernatant was filtrated through Whatman filter paper (Grade 1: 11 μm, VWR), and the volume was adjusted to 10 mL with the extraction buffer and then filtered again using a syringe filter (RC 0.45 μm, Macherey‐Nagel, Düren, Germany).

In order to concentrate and purify the vitamin B_12_, the plant extracts of the two replicates for each treatment and growth medium were pooled and cleansed using an Easi‐Extract Vitamin B_12_ immunoaffinity column (R‐Biopharm, Pfungstadt, Deutschland) according to the manufacturer's instructions. Briefly, the buffer in the immunoaffinity column was drained, after which 20 mL of plant extract for each treatment (10 mL from each of the two replicates) was passed through the column. The column was washed with 10 mL of MilliQ water and dried by passing air. Vitamin B_12_ was then eluted with 3 mL of methanol. The eluate was evaporated overnight at 65 °C, and the residue was reconstituted in 300 μL of MilliQ water acidified with 0.25 mL L^−1^ trifluoroacetic acid (pH 2.6).

The analysis of vitamin B_12_ content in the plant extract was performed with the same method used for bacterial cells with few modifications. For each replicate, the volume was 100 μL and was injected twice. The cyanocobalamin standards were eluted in MilliQ water acidified with 0.25 mL L^−1^ trifluoroacetic acid (pH 2.6), and the concentrations for setting the calibration curve were 0, 0.01, 0.02, 0.03, 0.05, 0.1, 0.2, and 0.3 ng μL^−1^.

### Colonization assessment

A cultivation‐based approach was used to confirm the colonization of lettuce plants by vitamin B_12_‐producing endophytic bacteria. For each replicate, including the negative control, the seedlings were removed gently from the MS medium and surface sterilized by stirring them for 1 min in 700 mL L^−1^ ethanol, followed by 2 min in 20 mL L^−1^ hypochlorite and they were rinsed three times in sterile MilliQ water for 1 min. At the end of the surface sterilization process, 100 μL of the water used for the third rinse was inoculated on tryptic soy agar (TSA) media to confirm the absence of bacteria on the plant surface. Then, 1 g of plant material and 9 mL of 9 g L^−1^ NaCl were mixed and homogenized with an Ultra‐Turrax homogenizer for 1 min. Each extract was diluted 1:100, and 100 μL were inoculated on TSA media and on TSA media supplemented with Pseudomonas CFC Selective Supplement (Merk), which were incubated at 27 °C for 48 h. Ten colonies presenting a similar morphology to the original strain used for seed treatment were selected for each replicate and grown overnight in tryptic soy broth (TSB) (Merck) medium. Subsequently, DNA isolation was performed using Nexttec 1^−Step^ DNA Isolation Kit for bacteria following manufacturer instructions.

The intergenic spacer (IGS region) of the rRNA gene was amplified using the primers pHr (5′‐ TGCGGCTGGATCACCTCCTT‐3′) and P23SR01 (5′‐ GGCTGCTTCTAAGCCAAC‐3′).^57^

Each amplification reaction was conducted in a final volume of 50 μL containing 1x Reaction Buffer BD (Solis BioDyne, Tartu, Estonia), 2.5 mM MgCl_2_ (Solis BioDyne), 0.2 mM dNTPs (Thermo Scientific, Waltham, Massachusetts, U.S), 0.3 mM of each primer, 2 U FIREPol DNA Polymerase (Solis BioDyne) and 4 μL of gDNA. The thermal cycling parameters were as follows: an initial denaturation step at 94 °C for 5 min, followed by 30 cycles of 45 s at 95 °C, 60 s at 54 °C and 2 min at 72 °C, and a final step of 10 min at 72 °C. The amplified DNA fragments were analyzed in 10 g L^−1^ agarose gels and visualized under UV light using DNA Marker Lambda/Hind III (A&A Biotechnology, Gdansk, Poland) as a reference.

Aliquots of 5 μL of PCR products were individually subjected to restriction fragment length polymorphism (RFLP) analysis without further purification. The digestion was performed for 3 h at 37 °C in a final volume of 15 μL containing 1× Tango buffer (Thermo Scientific) and 5 U of restriction endonuclease *Alu*I or *Hha*I (Thermo Scientific) with recognizing sequences 5′‐AG↓CT‐3′ and 5′‐GCG↓C‐3′ respectively. The restriction fragments were separated by electrophoresis on 25 g L^−1^ agarose gels and visualized under UV light using GeneRuler DNA Ladder Mix (Thermo Scientific) as a reference. The RFLP patterns obtained from bacteria isolated from lettuce were compared with those obtained from the parent strains. Thus, it was possible to establish whether the microorganisms belonged to the same strain.

## RESULTS

### Genome annotation and feature detection

The RAST server was used to annotate 65 genomes of endophytes from the AIT strain collection and the *Pseudomonas denitrificans* ATCC 13867 reference genome. After the annotation, the presence and completeness of the vitamin B_12_ metabolic pathway were evaluated for each genome. It was observed that 34 strains, including the ATCC 13867, had the vitamin B_12_ metabolic pathway in their genome with different degrees of completeness. These 34 strains were selected for further testing. Of the eight genes considered (Fig. 1), 22 strains had all of them, including the reference strain ATCC 13867; seven strains had seven genes, and five strains had six genes. No genes were detected in the remaining strains. The least frequent gene was the corrinoid adenosyltransferase (EC 2.5.1.17), which is present in only 23 genomes. The gene encoding adenosylcobalamin‐5‐P phosphatase (EC 3.1.3.73), the last enzyme of the pathway that transforms the adenosylcobalamin 5′‐phosphate into adenosylcobalamin, was present in 29 genomes. The enzyme threonine‐phosphate decarboxylase (EC 4.1.1.81) was found in 33 genomes, whereas the other five genes (EC 6.3.5.10, EC 6.3.1.10, EC 2.7.7.62, EC 2.4.2.21, EC 2.7.8.26) were present in 34 genomes.

The Bakta annotation tool was subsequently used to integrate and refine the analysis performed with RAST. In particular, this approach allowed the detection of the gene EC 2.5.1.17 in nine additional strains of the 34 selected based on the RAST analysis, increasing the number of strains carrying this gene from 23 to 32. The genes EC 3.1.3.73 and EC 4.1.1.81 were not found in any additional strains.

### Bacterial vitamin B_12_ analysis

**Figure 2:**
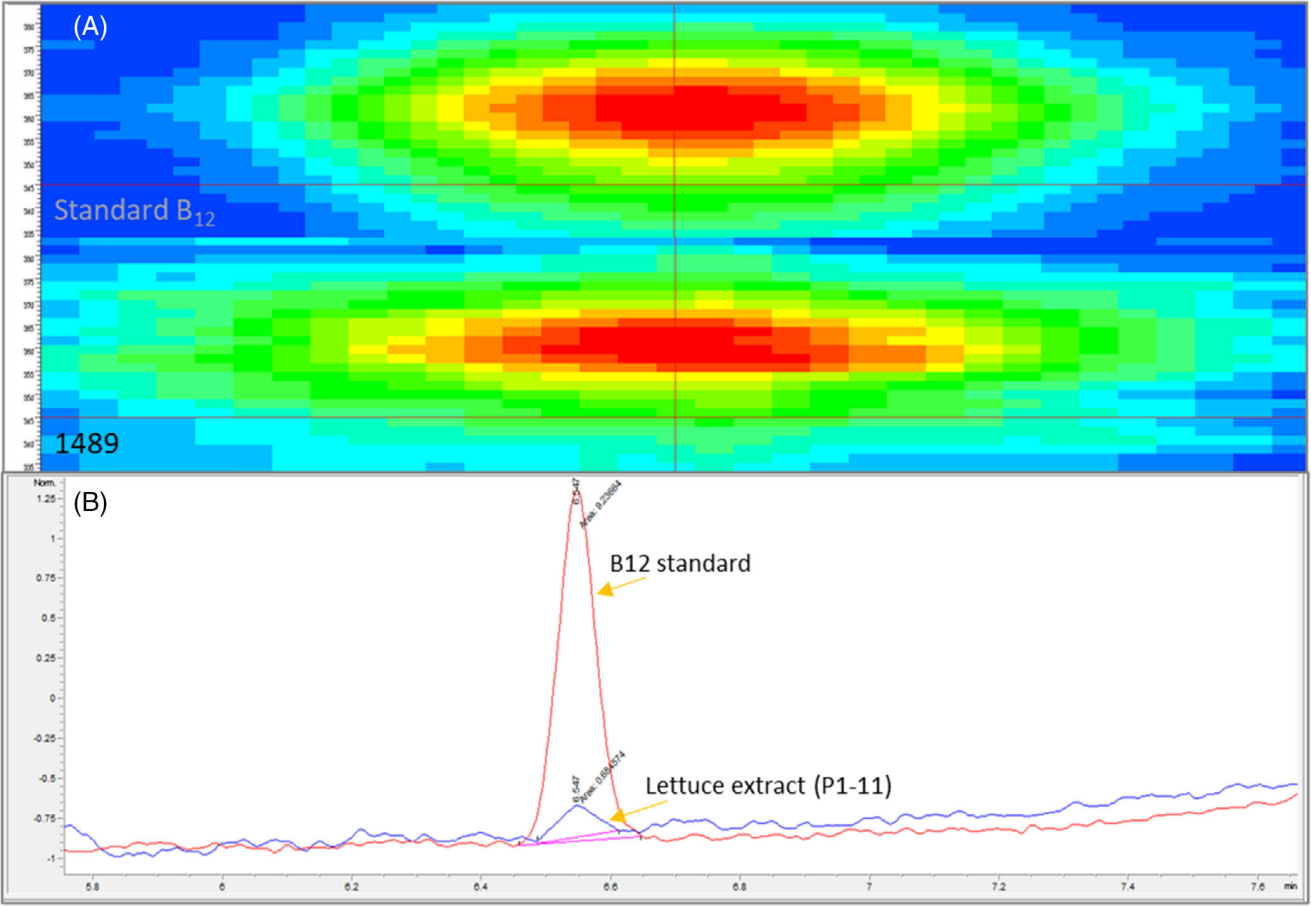
(A) Isoplots representing the 330–390 nm spectra from the pure vitamin B_12_ standard (top) and the extract of the bacterial strain *Pseudomonas* sp. 1489 (bottom). The isoplots depict the intensity of the peak according to the time (*x*) and the wavelength (*y*). (B) Comparison of chromatograms obtained from the immunoaffinity purified extracts of the vitamin B_12_ standard solution and the extract of lettuce plantlets treated with *Methylobacterium* sp. P1‐11 and grown on Murashige and Skoog (MS) medium supplemented with cobalt chloride.

The vitamin B_12_ production of the 34 strains selected based on the preliminary RAST analysis was tested by HPLC‐DAD analysis of the extracts obtained from pure bacterial cultures grown on LB broth supplemented with 0.018 g L^−1^ CoCl_2_ (Table 1). It was possible to detect and quantify vitamin B_12_ in 11 of the 34 strains tested. Production ranged from 1.067 to 6.438 μg of vitamin B_12_ per g of bacterial cells. The reference strain, *Pseudomonas denitrificans* ATCC 13867, was the least productive under tested conditions. For all identified peaks, the 330–390 nm spectra corresponded to that of the standard (Fig. 2(A)), confirming the specificity of the detected signal. The 11 producing strains belong to seven different genera, and in all of them, except for *Paenibacillus amylolyticus* 2136, the final eight genes of the metabolic pathway were detected using RAST‐based analysis. Subsequent refinement of the genome analysis with the Bakta annotation tool revealed the presence of eight analyzed genes in all producing strains.

**Table 1:** Bacterial strains showing vitamin B_12_ production and respective concentration expressed as μg of vitamin B_12_ for gram of bacterial cells

Strain name	Taxon	AIT‐ID	Number of genes	Source	μg B12/g cells
**ATCC 13867**	***Pseudomanas denitrificans***	**‐**	**8**	**‐**	**1.067**
F1_10	*Pseudomonas sp*.	1489	8	*Solanum tuberosum*	6.438
P1‐11	*Methylobacterium sp*.	1940	8	*Solanum tuberosum*	4.300
LZA 5–1	*Paenibacillus amylolyticus*	2136	7	*Triticum aestivum*	3.990
C8BA17	*Aneurinibacillus migulanus*	4261	8	*Allium schoenoprasum*	3.537
A3‐14	*Priestia aryabhattai*	4289	8	*Glycine max*	2.485
C13BA17	*Lysinibacillus fusiformis*	4265	8	*Allium schoenoprasum*	2.071
M3‐23	*Priestia megaterium*	1390	8	*Glycine max*	1.985
G66BA17	*Peribacillus frigoritolerans*	4238	8	*Lolium perenne*	1.797
T1_3	*Pseudomonas atacamensis*	1173	8	*Solanum tuberosum*	1.447
G65BA17	*Peribacillus frigoritolerans*	4237	8	*Lolium perenne*	1.184

For each strain, the table provides the strain name, species name, AIT strain collection ID, number of genes detected from the common vitamin B_12_ metabolic pathway (eight genes, Fig. 1), original isolation matrix, and vitamin B_12_ production (μg per gram of bacterial cells).

### Plant growth and vitamin B_12_ content

**Figure 3:**
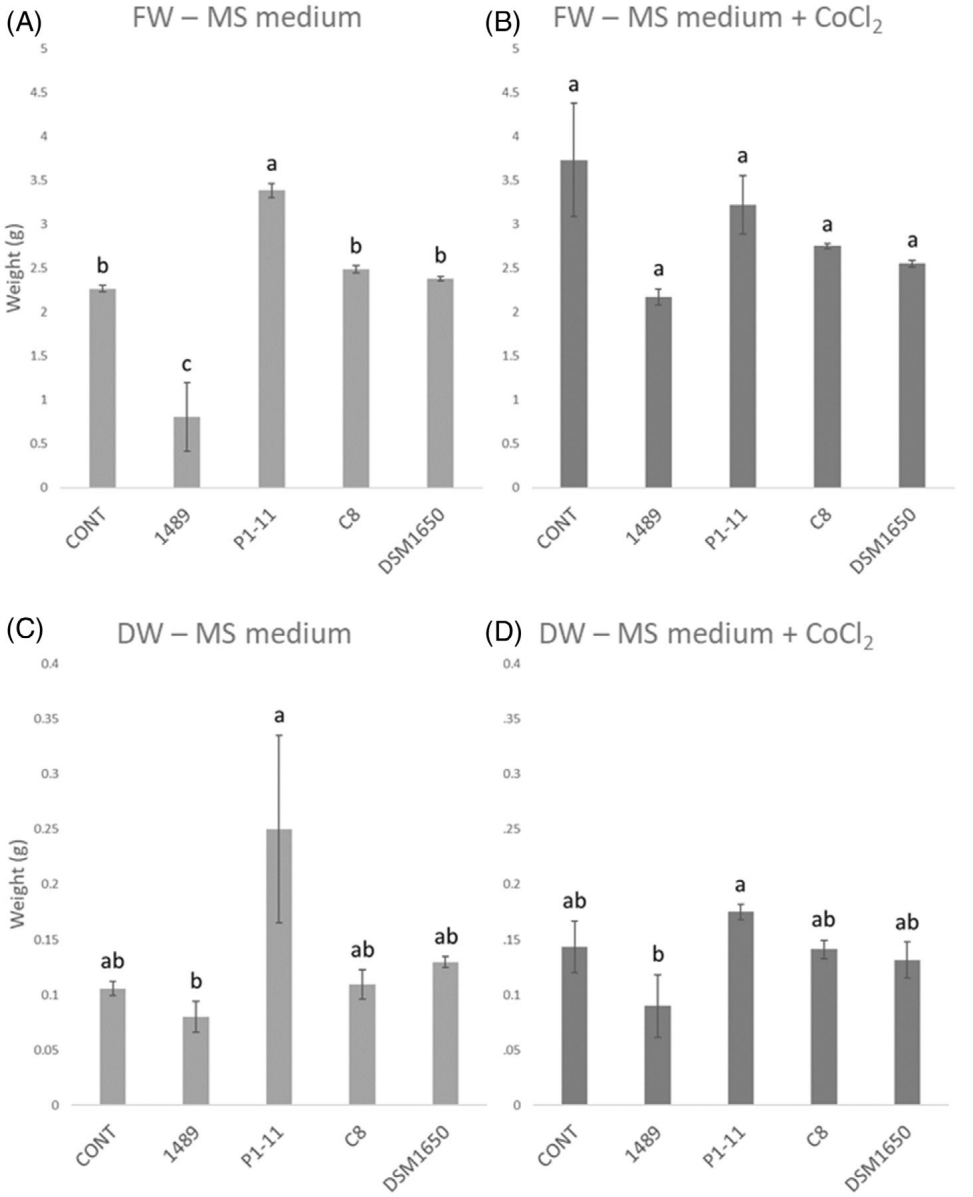
Average fresh weight values of epigean part of lettuce plants ± standard deviation (ten plant pools, two replicates) grown on Murashige and Skoog (MS) medium (A) and on MS medium supplemented with cobalt chloride (B). Average dry weight values of epigean part of lettuce plants ± standard deviation (ten plant pools, two replicates) grown on MS medium (C) and on MS medium supplemented with cobalt chloride (D). Different letters above the histograms resulting from Tukey's honest significant difference (HSD) test after analysis of variance (ANOVA) represent significant differences between treatments within the same medium. CONT – uninoculated control, 1498 – plants inoculated with *Pseudomonas sp*. 1489, P1‐11 – plants inoculated with *Methylobacterium sp*. P1‐11, C8 – plants inoculated with *Aneurinibacillus sp*. C8BA17, ATCC13867 – plants inoculated with *Pseudomonas denitrificans* ATCC 13867.

The three best vitamin B_12_ producers *in vitro* (*Pseudomonas* sp. 1489, *Methylobacterium* sp. P1‐11 and *Aneurinibacillus migulanus* C8BA17) possessing all eight metabolic pathway genes considered based on the RAST analysis, and the reference strain *Pseudomonas denitrificans* ATCC 13867, were selected to evaluate their efficacy in producing vitamin B_12_ in lettuce plants. Before proceeding with vitamin B_12_ extraction, the epiphytic part of the lettuce seedlings was weighted to evaluate the effect of bacteria on plant growth and ensure there were no negative effects on the yield. *Pseudomonas* sp. 1489 caused an average decrease in fresh weight of 64.5% compared with the control (*P* = 0.0011), and *Methylobacterium* sp. P1‐11 caused an average fresh weight gain of 49.5% compared with the control (*P* = 0.0045) (Fig. 3). In both cases, the difference was detected in plants grown on MS medium without the CoCl_2_ supplementation. With regard to the measurement of the dry weight, none of the bacterial strains tested caused a significant variation.

The potential plant growth‐promoting traits (PGPT) of *Methylobacterium* sp. P1‐11 were investigated using the web resource PLaBAse with the PGPT‐Pred module.

The submitted genome showed 3132 alignments to known proteins associated with PGPTs. Most of these proteins (~66%) were associated with indirect PGP mechanisms, such as stress control and biocontrol, competitive exclusion, colonization of plant system and stimulation of plant immune response. The remaining proteins (~34%) were involved in direct PGP mechanisms, which can be grouped into bioremediation, biofertilization and phytohormones and plant signal production (Supporting Information, Fig. S2).

The vitamin B_12_ extracts were analyzed by HPLC‐DAD after purification with immunoaffinity columns, and the results were expressed as μg of vitamin B_12_ per g of dry weight (DW) of lettuce leaves. With this technique, it was possible to detect vitamin B_12_ only in lettuce plants treated with *Methylobacterium* sp. P1‐11 (originally isolated from *Solanum tuberosum* ^58^ and previously shown to harbor genes of the vitamin B_12_ metabolic pathway^59^), grown both on MS medium and MS medium supplemented with CoCl_2_ (Fig. 2(B)). The amounts of vitamin B_12_ detected were 1.654 and 2.559 μg g^−1^, respectively.

### Colonization assessment

To confirm the colonization of lettuce plants by vitamin B_12_‐producing endophytes, bacterial isolation from lettuce leaves was performed, followed by gDNA extraction, intergenic spacer polymerase chain reaction (IGS PCR) amplification and RFLP digestion with two different restriction enzymes (*Alu*I and *Hha*I). In general, the enzymatic digestion of the IGS‐PCR amplification product provides a restriction pattern for each endonuclease that is conserved among the strains of the same species.^60^ Bacteria isolated from lettuce gave an identical pattern to the inoculated strain with both enzymes, *Alu*I and *Hha*I (Supporting Information, Fig. S3), except for *Aneurinibacillus migulanus* C8BA17 inoculated samples, from which the inoculated strain could not be retrieved under applied experimental conditions. The test therefore enabled us to confirm that the bacteria *Pseudomonas* sp. 1489, *Methylobacterium* sp. P1‐11 and *Pseudomonas denitrificans* ATCC 13867 colonized lettuce plants. It is, therefore, possible to hypothesize that the vitamin B_12_ present in lettuce plants treated with *Methylobacterium* sp. P1‐11 was actually produced by the inoculated bacteria.

## DISCUSSION

In recent years, people have become increasingly attentive to the problems caused by ongoing climate change, the challenges related to global population growth and the role that food production plays within this framework. The production of food of animal origin generates large emissions of greenhouse gases, contributing to global warming. There is a broad consensus that a transition towards more plant‐based diets can enhance the sustainability of our food systems.^61^, ^62^, ^63^, ^64^, ^65^ A plant‐based diet promotes several health benefits, such as reducing various risks related to chronic diseases and an overall increase in health and longevity.^66^, ^67^, ^68^, ^69^ However, strictly plant‐based diets pose a risk of vitamin B_12_ deficiency, as foods of plant origin are not a source of vitamin B_12_.^24^, ^25^ A solution to this problem could be offered through biofortification through vitamin B_12_ producing bacteria. Endophytes are already known to be capable of increasing the content of minerals or other nutrients in plants;^38^, ^39^, ^40^, ^42^, ^43^, ^70^, ^71^, ^72^, ^73^, ^74^ it is therefore feasible to extend their possible application for biofortification with additional nutraceutical compounds.

To screen the 66 genomes used in this study, the annotation obtained with the RAST server was compared with the prediction of enzymes involved in the metabolic pathways provided by the MetaCyc database and described by Balabanova *et al*.^16^ Rapid Annotations using Subsystems Technology was developed in 2008 to annotate bacterial and archaeal genomes. It supplies a standard pipeline for the identification and annotation of genomic features such as protein‐coding genes and RNA.^75^ The MetaCyc database and information on the structure of the vitamin B_12_ pathway^16^ were used to determine which genes belonging to the vitamin B_12_ metabolic pathway should be considered. It was decided to search each genome for the last eight genes involved in the vitamin B_12_ pathway as they are common to both the aerobic and the anaerobic vitamin B_12_ pathways.

More than half of the genomes analyzed (34 out of 66) had at least some genes involved in the vitamin B_12_ pathway, yet only 22 had all eight analyzed genes. Nevertheless, all 34 strains were selected for the *in vitro* experiments because the metabolic pathways could sometimes lack some genes or have unknown alternative genes.^12^, ^76^

The screening in this study also included much biodiversity. The 66 analyzed strains belonged to ten different genera, *Bacillus* (26), *Paenibacillus* (12), *Pseudomonas* (11, including the reference strain), *Pantoea* (4), *Priestia* (4), *Lysinibacillus* (2), *Peribacillus* (2), *Methylobacterium* (1), *Metabacillus* (1), *Aneurinibacillus* (1), *Kocuria* (1), and *Tardiphaga* (1). The stains that had at least six of the eight genes considered (through RAST analysis) belonged to the genera *Pseudomonas* (11, including the reference strain), *Paenibacillus* (11), *Priestia* (4), *Peribacillus* (2), *Lysinibacillus* (2), *Aneurinibacillus* (1), *Metabacillus* (1), *Methylobacterium* (1) and *Tardiphaga* (1). Finally, the strains that were confirmed as vitamin B_12_ *de novo* producers through HPLC‐DAD analysis, belonged to genera *Pseudomonas* (3, including the reference strain), *Peribacillus* (2), *Priestia* (2), *Paenibacillus* (1), *Lysinibacillus* (1), *Methylobacterium* (1), and *Aneurinibacillus* (1).

Based on the results obtained with RAST, of the 11 bacterial strains that were shown to be able to synthesize vitamin B_12_ *de novo*, ten had all eight genes of the vitamin B_12_ metabolic pathway considered in the analysis. In contrast, the strain *Paenibacillus amylolyticus* 2136, only had seven. The gene missing in *P. amylolyticus* 2136, which was also missing in all the other ten *Paenibacillus* strains analyzed, was the gene encoding for the enzyme corrinoid adenosyltransferases (CobA/CobO, EC 2.5.1.17) (Fig. 1), whose function is the adenosylation of cob(I)yrinic acid a,c‐diamide to cobalt ion, resulting in adenosylcobyrinic acid a,c‐diamide.^14^, ^77^ To explain how the strain *P. amylolyticus* 2136 was able to produce vitamin B_12_ despite apparently lacking a gene, the Bakta gene annotation tool was used to complement the analysis performed with RAST. With this approach, the EC 2.5.1.17 gene was found in eight of the 12 paenibacilli analyzed, including P. amylolyticus 2136.

We also found differences in the potential to produce vitamin B_12_ among the different genera. Remarkably, even though we analyzed only one strain of *Lysinibacillus* sp., *Methylobacterium* sp., and *Aneurinibacillus* sp., all of these were capable of producing vitamin B_12_. Thus, it could suggest that these genera could be a promising source of vitamin B_12_‐producing bacteria. In fact, *Methylobacterium* spp. have been known for a long time to be vitamin B_12_ producers,^78^ and there are some studies demonstrating that *Lysinibacillus* strains have the essential genes for vitamin B_12_ synthesis, but it has not been experimentally proven that they can produce it *de novo*.^79^, ^80^ No studies were found regarding the ability of *Aneurinibacillus* strains to synthesize vitamin B_12_; however, bacterial strains belonging to this species have been reported to be endophytes.^81^, ^82^ It would therefore be interesting to screen more bacterial strains of these genera, both *in silico* and *in vitro*.

The proportion of vitamin B_12_‐producing *Pseudomonas* strains was also quite considerable (⁓27%). However, this outcome is not surprising as *Pseudomonas* strains are already known vitamin B_12_ producers and are widely exploited in the industry for its production.^16^

The species *Peribacillus frigoritolerans, Priestia aryabhattai*, and *Priestia megaterium* were classified as *Bacillus* until 2020.^83^, ^84^ *Priestia megaterium*, formerly *Bacillus megaterium*, has been employed as an industrial producer of vitamin B_12_ through the anaerobic pathway.^85^, ^86^, ^87^

On the other hand, many strains containing the metabolic pathway (23 out of 34) did not show the ability to produce vitamin B_12_ under the tested conditions. This could be explained by the fact that only the last eight genes of the pathway, shared by both the aerobic and the anaerobic pathways, were considered in the genomic analysis. It could therefore be hypothesized that the strains that have the eight shared genes but have not produced vitamin B_12_ may be anaerobic producers, and consequently, the aerobic experimental conditions did not allow them to produce vitamin B_12_ *de novo*. This could also be the case for the 12 paenibacilli analyzed in this study. *In silico*, seven had seven genes, four had six genes, and one had none of the genes considered, but, out of the 11 paenibacilli tested *in vitro*, only one (*P. amylolyticus* 2136) was able to produce vitamin B_12_ under the aerobic experimental conditions. Previous studies have demonstrated that *Paenibacillus* strains have the genes essential for vitamin B_12_ synthesis. However, it has not been experimentally proven whether and under which conditions they can produce vitamin B_12_.^80^, ^88^, ^89^ Moreover, *Paenibacillus* strains have been reported to be facultative anaerobic,^90^ thus they may need anaerobic conditions for vitamin B_12_ production.

Even though the oxygen effect provides the most straightforward way to explain the observed differences between the genomic potential and the actual, observed vitamin B_12_ production, it is not the only one. This becomes evident when reflecting on the genus *Pseudomonas* and considering seven strains that did not produce a detectable amount of vitamin B_12_ despite having all eight genes considered, although the industrial production of vitamin B_12_ through *Pseudomonas* strains always takes place under aerobic conditions.^16^, ^91^, ^92^ Vitamins are complex molecules with intricate synthetic pathways that are highly regulated and co‐depend on many external factors; thus, optimized growth conditions are essential for efficient biosynthesis.^1^ Indeed, the optimal conditions used in the industrial process for aerobic vitamin B_12_ production through *P. denitrificans* are significantly more customized than those applied in our study. The industrial process takes place at 30 °C, with a pH of 6.0–7.0 in 120 m^3^ fermenters and lasts about 6–7 days. Sucrose is used as a carbon and yeast extract as a nitrogen source, mineral salts are also added.^93^ Fermentation begins with a high level of dissolved oxygen concentration (8–10%) followed by its reduction to 2–5% (49–106 h) and further below 2% (107–168 h).^94^, ^95^ This multi‐stage dissolved oxygen concentration (DOC) control strategy increases the vitamin B_12_ yield by approximately 20% (70 mg L^−1^). At the beginning of the culture growth, the medium is supplemented with 10–25 mg L^−1^ of 5,6‐dimethylbenzimidazole (DMB), a precursor of vitamin B_12_, and 40–200 mg L^−1^ cobalt‐nitrate.^96^ As previously described, our experimental conditions were drastically different. For example, we did not design a multi‐stage DOC control strategy or add any vitamin B_12_ precursor to the medium. This choice was made because our objective was not to look for a producer of vitamin B_12_ to be included in an industrial production process but a strain capable of producing vitamin B_12_ in a completely independent way, which could, therefore, have a better chance of producing vitamin B_12_ in plants. Nevertheless, considering that all of our producers showed higher yields of vitamin B_12_ than the reference strain, their exploitation for industrial production of vitamin B_12_ could be further explored.

Vitamin B_12_ extracted from lettuce was quantified by HPLC‐DAD and was expressed as μg of vitamin B_12_ per g of dry weight (DW) of lettuce leaves. Depending on the cultivation medium (with or without cobalt supplementation) lettuce treated with *Methylobacterium* sp. P1‐11 had vitamin B_12_ content of 2.559 μg g^−1^ and 1.654 μg g^−1^, respectively. Considering that 1 g of dry weight corresponds to about 20 g of fresh lettuce, we can extrapolate that a normal portion of lettuce (80 g), would contain sufficient amounts of vitamin B_12_ to fulfil recommended daily allowance (RDA = 2.4 μg per day; one portion of lettuce = 6.6–10.2 μg). This finding could lead to the development of a plant‐based solution to vitamin B_12_ deficiency that could affect people with a vegan diet. However, we have no further information on the bioavailability of the vitamin B_12_ detected in lettuce, nor do we know which forms of vitamin B_12_ were produced by *Methylobacterium* sp. P1‐11 because our extraction method utilized KCN, which brings all the vitamin B_12_ analogues to the more stable cyanocobalamin. In light of these considerations, we recognize the need for further studies to address this lack of knowledge; however, we believe that our work provides an excellent proof of concept of how microorganisms can be used to fortify lettuce, or more generally edible plants, with vitamin B_12_.

Although it was not the aim of the study to prove that the vitamin B_12_‐producing strains had plant‐growth‐promoting properties, it was important to verify that they do not cause a lettuce yield reduction. For this reason, fresh and dry weight were measured and statistically evaluated. Although we are aware of the size effect on the statistical power of the significance tests,^97^ we decided to include only two replicates in the growth evaluation experiment and consequently in the ANOVA. This choice was made because the experiment was only made to verify that the bacteria did not negatively affect lettuce yield – their PGPTs were not the focus of our work. We not only verified that the *Methylobacterium* sp. P1‐11 did not have a negative effect on plant growth, but we even observed that it had a significant positive effect on fresh weight.

In order to provide an explanation and a stronger support for the observed positive effect of *Methylobacterium* sp. P1‐11 on lettuce growth, we decided to investigate its genomic protein sequences through PLaBAse, a valuable web resource for the prediction of plant‐associated microbial genes, and, specifically, for the prediction of genes that promote plant growth through the use of the PGPT‐Pred tool.^55^, ^98^ The PLaBAse investigation identified over 3000 proteins associated with PGPTs that could have contributed to the growth enhancement of lettuce plants by direct and indirect mechanisms. It is also particularly interesting to note that 25% of the proteins associated with PGPTs by PLaBAse are related to plant colonization. This reinforces what has already been observed about the high ability of *Methylobacterium* sp. P1‐11 to colonize lettuce seedlings and thus introduce significant amounts of vitamin B_12_ into them.

We could not detect any vitamin B_12_ synthesized *in planta* by the other strains tested, even though their presence was confirmed through a cultivation‐based approach combined with IGS‐RFLP typing for all of them except *Aneurinibacillus migulanus* C8BA17. However, successful colonization is only the first step towards establishing potentially beneficial symbiosis. The interactions between endophytes and their host are complex and not fully understood.

Although sufficient expression was observed under the experimental conditions, from a future perspective, it would be important to gain a deeper understanding of the regulatory mechanisms and environmental factors that control vitamin B_12_ production by the identified bacterial strain. Further research is also needed to explore the multidimensional network between plants, endophytes, and their environment, and to better exploit the potential of these microorganisms for sustainable agriculture or for large‐scale production of host metabolites, which have potential applications in various fields.^99^, ^100^

## CONCLUSION

We identified several bacteria strains capable of synthesizing vitamin B_12_, one of which produced vitamin B_12_ in lettuce plants under defined experimental conditions. To our knowledge, this is the first time a bacterial endophyte was used for this purpose. This work therefore provides evidence that bacterial endophytes could be utilized to enhance the nutraceutical values of plant‐based foods. Furthermore, the applied bioinformatic‐based screening approach represents promising potential for fast identification of potential biofortification candidates from existing strain collections.

## CONFLICTS OF INTEREST

The authors declare that they have no conflicts of interest.

## Supporting information


**Fig. S1.** Structure of cobalamin. R^a^ = deoxyadenosine which forms adenosylcobalamin (AdoCbl); R^b^ = cyano group which forms cyanocobalamin (CNCbl); R^c^ = deoxyadenosine which forms methylcobalamin (MeCbl), R^d^ = hoxidrile group which forms hydroxycobalamin (OHCbl). Designed with www.reaxys.com.
**Fig. S2**. Krona plot representation of the major PGPTs found in Methylobacterium sp. P1‐11, generated with PLaBase web tool.
**Fig. S3**. IGS‐RFLP patterns (agarose gel 2.5%) obtained from Methylobacterium sp. P1‐11 and from bacteria isolated from lettuce (Let1‐Let10) treated with this strain. The patterns were obtained using AluI (left)and HhaI (right) endonucleases.

**Table S1.** All the 65 bacterial endophytic strains from the Bioresources strain collection of the AIT Austrian Institute of Technology, selected for this study. For each strain are reported, the AIT strain collection ID, the scientific name, the strain name, the matrix from where it was isolated, the genome length, its completeness, and the percentage of GC.

## Data Availability

The whole genome sequence data and associated metadata generated for this study are available in the NCBI database under the BioProject PRJNA1072664. An exception is strain P1‐11, which was previously uploaded under the BioProject PRJNA393298 and assigned to the BioSample SAMN07447406.

## References

[1] Acevedo‐Rocha CG , Gronenberg LS , Mack M , Commichau FM and Genee HJ , Microbial cell factories for the sustainable manufacturing of B vitamins. Curr Opin Biotechnol 56:18–29 (2019). 10.1016/j.copbio.2018.07.006. 30138794

[2] O’leary F and Samman S , Vitamin B 12 in health and disease. Nutrients 2:299–316 (2010). 10.3390/nu2030299. 22254022 PMC3257642

[3] Takahashi‐Iñiguez T , García‐Hernandez E , Arreguín‐Espinosa R and Flores ME , Role of vitamin B 12 on methylmalonyl‐CoA mutase activity. J Zhejiang Univ, Sci, B 13:423–437 (2012). 10.1631/jzus.B1100329. 22661206 PMC3370288

[4] Nouri A , Patel K , Montejo J , Nasser R , Gimbel DA , Sciubba DM *et al*., The role of vitamin B12 in the management and optimization of treatment in patients with degenerative cervical myelopathy. Global Spine J 9:331–337 (2018). 10.1177/2192568218758633. 31192102 PMC6542160

[5] Froese DS , Fowler B and Baumgartner MR , Vitamin B12, folate, and the methionine remethylation cycle – biochemistry, pathways, and regulation. J Inherit Metab Dis 42:673–685 (2019). 10.1002/jimd.12009. 30693532

[6] Institute of Medicine (US) Standing Committee on the Scientific Evaluation of Dietary Reference Intakes and its Panel on Folate OBV and C , Dietary Reference Intakes for Thiamin, Riboflavin, Niacin, Vitamin B6, Folate, Vitamin B12, Pantothenic Acid, Biotin, and Choline. National Academic Press, Washington, DC (1998). 10.17226/6015. 23193625

[7] Carmel R , Prevalence of undiagnosed pernicious anemia in the elderly. Arch Intern Med 156:1097–1100 (1996). 8638997

[8] Hernandez CMR and Oo TH , Advances in mechanisms, diagnosis, and treatment of pernicious anemia. Discovery Med 19:159–168 (2015). 25828519

[9] Pawlak R , James PS , Raj S , Cullum‐Dugan D and Lucus D , Understanding Vitamin B12. Am J Lifestyle Med 7:60–65 (2012). 10.1177/1559827612450688.

[10] Langan RC and Goodbred AJ , Vitamin B12 deficiency: recognition and management. Am Fam Physician 96:384–389 (2017). 28925645

[11] Danchin A and Braham S , Coenzyme B12 synthesis as a baseline to study metabolite contribution of animal microbiota. J Microbial Biotechnol 10:688–701 (2017). 10.1111/1751-7915.12722. PMC5481537 28612402

[12] Shelton AN , Seth EC , Mok KC , Han AW , Jackson SN , Haft DR *et al*., Uneven distribution of cobamide biosynthesis and dependence in bacteria predicted by comparative genomics. ISME J 13:789–804 (2018). 10.1038/s41396-018-0304-9. 30429574 PMC6461909

[13] Lu X , Heal KR , Ingalls AE , Doxey AC and Neufeld JD , Metagenomic and chemical characterization of soil cobalamin production. ISME J 14:53–66 (2019). 10.1038/s41396-019-0502-0. 31492962 PMC6908642

[14] Bryant DA , Hunter CN and Warren MJ , Biosynthesis of the modified tetrapyrroles – the pigments of life. J Biol Chem 295:6888–6925 (2020). 10.1074/jbc.REV120.006194. 32241908 PMC7242693

[15] Fang H , Kang J and Zhang D , Microbial production of vitamin B12: a review and future perspectives. Microb Cell Fact 16:15 (2017). 10.1186/s12934-017-0631-y. PMC5282855 28137297

[16] Balabanova L , Averianova L , Marchenok M , Son O and Tekutyeva L , Microbial and genetic resources for cobalamin (vitamin b12) biosynthesis: from ecosystems to industrial biotechnology. International journal of molecular. Science 22:4522 (2021). 10.3390/ijms22094522. PMC8123684 33926061

[17] González‐Montaña JR , Escalera‐Valente F , Alonso AJ , Lomillos JM , Robles R and Alonso ME , Relationship between vitamin B12 and cobalt metabolism in domestic ruminant: an update. Animals 10:1855 (2020). 10.3390/ani10101855. 33053716 PMC7601760

[18] Croft MT , Lawrence AD , Raux‐Deery E , Warren MJ and Smith AG , Algae acquire vitamin B12 through a symbiotic relationship with bacteria. Nature 438:90–93 (2005). 10.1038/nature04056. 16267554

[19] Helliwell KE , The roles of B vitamins in phytoplankton nutrition: new perspectives and prospects. New Phytol 216:62–68 (2017). 10.1111/nph.14669. 28656633

[20] Watanabe F , Yabuta Y , Tanioka Y and Bito T , Biologically active vitamin B12 compounds in foods for preventing deficiency among vegetarians and elderly subjects. J Agric Food Chem 61:6769–6775 (2013). 10.1021/jf401545z. 23782218

[21] Watanabe F , Schwarz J , Takenaka S , Miyamoto E , Ohishi N , Nelle E *et al*., Characterization of vitamin B12 compounds in the wild edible mushrooms black trumpet (Craterellus cornucopioides) and Golden Chanterelle (Cantharellus cibarius). J Nutr Sci Vitaminol 58:438–441 (2012). 10.3177/jnsv.58.438. 23419403

[22] Teng F , Bito T , Takenaka S , Yabuta Y , Shimomure N and Watanabe F , Determination and characterization of corrinoid compounds in truffle (tuber spp.) and shoro (Rhizopogon rubescens) fruiting bodies. Mushroom Sci Biotechnol 22:159–164 (2015).

[23] Burgess CM , Smid EJ and van Sinderen D , Bacterial vitamin B2, B11 and B12 overproduction: an overview. Int J Food Microbiol 133:1–7 (2009). 10.1016/j.ijfoodmicro.2009.04.012. 19467724

[24] Pawlak R , Parrott SJ , Raj S , Cullum‐Dugan D and Lucus D , How prevalent is vitamin B12 deficiency among vegetarians? Nutr Rev 71:110–117 (2013). 10.1111/nure.12001. 23356638

[25] Kumar SS , Chouhan RS and Thakur MS , Trends in analysis of vitamin B12. Anal Biochem 398:139–149 (2010). 10.1016/j.ab.2009.06.041. 19932677

[26] Nakos M , Pepelanova I , Beutel S , Krings U , Berger RG and Scheper T , Isolation and analysis of vitamin B12 from plant samples. Food Chem 216:301–308 (2017). 10.1016/j.foodchem.2016.08.037. 27596424

[27] Wall LG , The Actinorhizal Symbiosis. J Plant Growth Regul 19:167–182 (2000). 10.1007/s003440000027. 11038226

[28] Mitter B , Nter Brader G , Pfaffenbichler N and Sessitsch A , Next generation microbiome applications for crop production‐limitations and the need of knowledge‐based solutions. Curr Opin Microbiol 49:59–65 (2019). 10.1016/j.mib.2019.10.006. 31731227

[29] Liu J , Fimognari L , de Almeida J , Jensen CNG , Compant S , Oliveira T *et al*., Effect of bacillus paralicheniformis on soybean (Glycine max) roots colonization, nutrient uptake and water use efficiency under drought stress. J Agron Crop Sci 209:547–565 (2023). 10.1111/jac.12639.

[30] Faist H , Trognitz F , Antonielli L , Symanczik S , White PJ and Sessitsch A , Potato root‐associated microbiomes adapt to combined water and nutrient limitation and have a plant genotype‐specific role for plant stress mitigation. Environ Microbiome 18:1–19 (2023). 10.1186/s40793-023-00469-x. 36918963 PMC10012461

[31] Cappellari L d R , Santoro MV , Nievas F , Giordano W and Banchio E , increase of secondary metabolite content in marigold by inoculation with plant growth‐promoting rhizobacteria. Appl Soil Ecol 70:16–22 (2013). 10.1016/j.apsoil.2013.04.001.

[32] Yadav AN , Kumar R , Kumar S , Kumar V , Sugitha T , Singh B *et al*., Beneficial microbiomes: biodiversity and potential biotechnological applications for sustainable agriculture and human health. J Appl Biol Biotechnol 5:45–57 (2017). 10.7324/JABB.2017.50607.

[33] Yadav DR , Adhikari M , Kim SW , Kim HS and Lee YS , Suppression of fusarium wilt caused by Fusarium oxysporum f. sp. lactucae and growth promotion on lettuce using bacterial isolates. J Microbiol Biotechnol 31:1241–1255 (2021). 10.3390/jof9030336. 34373438 PMC9705851

[34] Maksimov IV , Veselova SV , Nuzhnaya TV , Sarvarova ER and Khairullin RM , Plant growth‐promoting bacteria in regulation of plant resistance to stress factors. Russ J Plant Physiol 62:715–726 (2015). 10.1134/S1021443715060114.

[35] Singh U , Praharaj CS , Singh SS and Singh NP , Biofortification: Introduction, approaches, limitations, and challenges. Biofortif Food Crops, Springer, New Deli:3–18 (2016). 10.1007/978-81-322-2716-8.

[36] White PJ and Broadley MR , Biofortification of crops with seven mineral elements often lacking in human diets‐‐iron, zinc, copper, calcium, magnesium, selenium and iodine. New Phytol 182:49–84 (2009). 10.1111/j.1469-8137.2008.02738.x. 19192191

[37] Hussain A , Zahir ZA , Asghar HN , Ahmad M , Jamil M , Naveed M *et al*., Zinc solubilizing bacteria for zinc biofortification in cereals: a step toward sustainable nutritional security, in Role of Rhizospheric Microbes in Soil: Volume 2: Nutrient Management and Crop Improvement. Springer, Singapore, pp. 203–227 (2018). 10.1007/978-981-13-0044-8_7.

[38] Kaur T , Rana KL , Kour D , Sheikh I , Yadav N , Kumar V *et al*., Microbe‐mediated biofortification for micronutrients: present status and future challenges. New and future developments in microbial. Biotechnol Bioeng:1–17 (2020). 10.1016/B978-0-12-820528-0.00002-8.

[39] Jha AB and Warkentin TD , Biofortification of pulse crops: status and future perspectives. Plan Theory 9:73 (2020). 10.3390/plants9010073. PMC7020478 31935879

[40] Gopalakrishnan S , Vadlamudi S , Samineni S and Sameer Kumar CV , Plant growth‐promotion and biofortification of chickpea and pigeonpea through inoculation of biocontrol potential bacteria, isolated from organic soils. SpringerPlus 5:1882 (2016). 10.1186/s40064-016-3590-6. PMC5082106 27833841

[41] Khanna K , Jamwal VL , Sharma A , Gandhi SG , Ohri P , Bhardwaj R *et al*., Supplementation with plant growth promoting rhizobacteria (PGPR) alleviates cadmium toxicity in Solanum lycopersicum by modulating the expression of secondary metabolites. Chemosphere 230:628–639 (2019). 10.1016/j.chemosphere.2019.05.072. 31128509

[42] Liu Z , Zhou J , Li Y , Wen J and Wang R , Bacterial endophytes from Lycoris radiata promote the accumulation of Amaryllidaceae alkaloids. Microbiol Res 239:126501 (2020). 10.1016/j.micres.2020.126501. 32585579

[43] Sun J , Chang M , Li H , Zhang Z , Chen Q , Chen Y *et al*., Endophytic bacteria as contributors to Theanine production in Camellia sinensis. J Agric Food Chem 67:10685–10693 (2019). 10.1021/acs.jafc.9b03946. 31479251

[44] Flores‐Félix JD , Silva LR , Rivera LP , Marcos‐García M , García‐Fraile P , Martínez‐Molina E *et al*., Plants probiotics as a tool to produce highly functional fruits: the case of Phyllobacterium and vitamin C in strawberries. PLoS One 10:e0122281 (2015). 10.1371/journal.pone.0122281. 25874563 PMC4398434

[45] Gurevich A , Saveliev V , Vyahhi N and Tesler G , QUAST: quality assessment tool for genome assemblies. Bioinformatics 29:1072–1075 (2013). 10.1093/bioinformatics/btt086. 23422339 PMC3624806

[46] Parks DH , Imelfort M , Skennerton CT , Hugenholtz P and Tyson GW , CheckM: assessing the quality of microbial genomes recovered from isolates, single cells, and metagenomes. Genome Res 25:1043–1055 (2015). 10.1101/gr.186072.114. 25977477 PMC4484387

[47] Chaumeil PA , Mussig AJ , Hugenholtz P and Parks DH , GTDB‐Tk: a toolkit to classify genomes with the genome taxonomy database. Bioinformatics 36:1925–1927 (2020). 10.1093/bioinformatics/btz848. PMC7703759 31730192

[48] Parks DH , Chuvochina M , Rinke C , Mussig AJ , Chaumeil PA and Hugenholtz P , GTDB: an ongoing census of bacterial and archaeal diversity through a phylogenetically consistent, rank normalized and complete genome‐based taxonomy. Nucleic Acids Res 50:D785–D794 (2022). 10.1093/nar/gkab776. 34520557 PMC8728215

[49] Camacho C , Coulouris G , Avagyan V , Ma N , Papadopoulos J , Bealer K *et al*., BLAST+: architecture and applications. BMC Bioinf 10:1–9 (2009). 10.1186/1471-2105-10-421. PMC2803857 20003500

[50] EFSA statement on the requirements for whole genome sequence analysis of microorganisms intentionally used in the food chain. EFSA J 22:e8912 (2024). DOI: 10.2903/j.efsa.2024.8912. PMC11317806 39135845

[51] Schwengers O , Jelonek L , Dieckmann MA , Beyvers S , Blom J and Goesmann A , Bakta: rapid and standardized annotation of bacterial genomes via alignment‐free sequence identification. Microb Genomes 7:000685 (2021). 10.1099/mgen.0.000685. PMC8743544 34739369

[52] Aziz RK , Bartels D , Best A , DeJongh M , Disz T , Edwards RA *et al*., The RAST server: rapid annotations using subsystems technology. BMC Genomics 9:1–15 (2008). 10.1186/1471-2164-9-75. 18261238 PMC2265698

[53] Ainala SK , Somasundar A and Park S , Complete genome sequence of Pseudomonas denitrificans ATCC 13867. Genome Announc 1:257–270 (2013). 10.1128/genomeA.00257-13. PMC3668002 23723394

[54] Krieger CJ , Zhang P , Mueller LA , Wang A , Paley S , Arnaud M *et al*., MetaCyc: a multiorganism database of metabolic pathways and enzymes. Nucleic Acids Res 32:D438–D442 (2004). 10.1093/nar/gkh100. 14681452 PMC308834

[55] Patz S , Gautam A , Becker M , Ruppel S , Rodríguez‐Palenzuela P and Huson DH , PLaBAse: a comprehensive web resource for analyzing the plant growth‐promoting potential of plant‐associated bacteria. bioRxiv (2021). 10.1101/2021.12.13.472471.

[56] Chamlagain B , Edelmann M , Kariluoto S , Ollilainen V and Piironen V , Ultra‐high performance liquid chromatographic and mass spectrometric analysis of active vitamin B12 in cells of Propionibacterium and fermented cereal matrices. Food Chem 166:630–638 (2015). 10.1016/j.foodchem.2014.06.068. 25053103

[57] Massol‐Deya AA , Odelson DA , Hickey RF and Tiedje JM , Bacterial community fingerprinting of amplified 16S and 16–23S ribosomal DNA gene sequences and restriction endonuclease analysis (ARDRA), in Molecular Microbial Ecology Manual. Springer, Netherlands, pp. 289–296 (1995). 10.1007/978-94-011-0351-0_20.

[58] del Barrio‐Duque A , Ley J , Samad A , Antonielli L , Sessitsch A and Compant S , Beneficial endophytic bacteria‐serendipita indica interaction for crop enhancement and resistance to Phytopathogens. Front Microbiol 10:499198 (2019). 10.3389/fmicb.2019.02888. PMC6930893 31921065

[59] del Barrio‐Duque A , Samad A , Nybroe O , Antonielli L , Sessitsch A and Compant S , Interaction between endophytic Proteobacteria strains and Serendipita indica enhances biocontrol activity against fungal pathogens. Plant Soil 451:277–305 (2020). 10.1007/s11104-020-04512-5.

[60] Quirós M , Martorell P , Valderrama MJ , Querol A , Peinado JM and de Silóniz MI , PCR‐RFLP analysis of the IGS region of rDNA: a useful tool for the practical discrimination between species of the genus Debaryomyces. Antonie Van Leeuwenhoek 90:211–219 (2006). 10.1007/s10482-006-9076-8. 16838194

[61] Aiking H and de Boer J , The next protein transition. Trends Food Sci Technol 105:515–522 (2020). 38620223 10.1016/j.tifs.2018.07.008 PMC7127173

[62] Willett W , Rockström J , Loken B , Springmann M , Lang T , Vermeulen S *et al*., Food in the anthropocene: the EAT–lancet commission on healthy diets from sustainable food systems. Lancet 393:447–492 (2019). 10.1016/S0140-6736(18)31788-4. 30660336

[63] Clark M and Tilman D , Comparative analysis of environmental impacts of agricultural production systems, agricultural input efficiency, and food choice. Environ Res Lett 12:064016 (2017). 10.1088/1748-9326/aa6cd5.

[64] Poore J and Nemecek T , Reducing food's environmental impacts through producers and consumers. Science 360(6):987–992 (2018). 10.1126/science.aaq0216. 29853680

[65] Godfray HCJ , Aveyard P , Garnett T , Hall JW , Key TJ , Lorimer J *et al*., Meat consumption, health, and the environment. Science 361:eaam5324 (2018). 10.1126/science.aam5324. 30026199

[66] Tonstad S , Butler T , Yan R and Fraser GE , Type of vegetarian diet, body weight, and prevalence of type 2 diabetes. Diabetes Care 32:791–796 (2009). 10.2337/dc08-1886. 19351712 PMC2671114

[67] Fraser GE , Vegetarian diets: what do we know of their effects on common chronic diseases? Am J Clin Nutr 89:1607S–1612S (2009). 10.3945/ajcn.2009.26736K. 19321569 PMC2677008

[68] Barnard ND , Scialli AR , Turner‐McGrievy G , Lanou AJ and Glass J , The effects of a low‐fat, plant‐based dietary intervention on body weight, metabolism, and insulin sensitivity. Am J Med 118:991–997 (2005). 10.1016/j.amjmed.2005.03.039. 16164885

[69] Appleby PN , Allen NE and Key TJ , Diet, vegetarianism, and cataract risk. Am J Clin Nutr 93:1128–1135 (2011). 10.3945/ajcn.110.004028. 21430115

[70] Singh D , Geat N , Rajawat MVS , Prasanna R , Kar A , Singh AM *et al*., Prospecting endophytes from different Fe or Zn accumulating wheat genotypes for their influence as inoculants on plant growth, yield, and micronutrient content. Ann Microbiol 68:815–833 (2018). 10.1007/s13213-018-1388-1.

[71] Singh D , Rajawat MVS , Kaushik R , Prasanna R and Saxena AK , Beneficial role of endophytes in biofortification of Zn in wheat genotypes varying in nutrient use efficiency grown in soils sufficient and deficient in Zn. Plant Soil 416:107–116 (2017). 10.1007/s11104-017-3189-x.

[72] Rana A , Kabi SR , Verma S , Adak A , Pal M , Shivay YS *et al*., Prospecting plant growth promoting bacteria and cyanobacteria as options for enrichment of macro‐ and micronutrients in grains in rice–wheat cropping sequence. Cogent Food Agric 1:1037379 (2015). 10.1080/23311932.2015.1037379.

[73] Durán P , Acuña JJ , Jorquera MA , Azcón R , Paredes C , Rengel Z *et al*., Endophytic bacteria from selenium‐supplemented wheat plants could be useful for plant‐growth promotion, biofortification and Gaeumannomyces graminis biocontrol in wheat production. Biol Fertil Soils 50:983–990 (2014). 10.1007/s00374-014-0920-0.

[74] Ku YS , Rehman HM and Lam HM , Possible roles of Rhizospheric and Endophytic microbes to provide a safe and affordable means of crop biofortification. Agronomy 9:764 (2019). 10.3390/agronomy9110764.

[75] Brettin T , Davis JJ , Disz T , Edwards RA , Gerdes S , Olsen GJ *et al*., RASTtk: a modular and extensible implementation of the RAST algorithm for building custom annotation pipelines and annotating batches of genomes. Sci Rep 5:1–6 (2015). 10.1038/srep08365. PMC4322359 25666585

[76] Torres AC , Vannini V , Font G , Saavedra L and Taranto MP , Novel pathway for Corrinoid compounds production in lactobacillus. Front Microbiol 9:2256 (2018). 10.3389/fmicb.2018.02256. 30319575 PMC6167548

[77] Caspi R , Billington R , Keseler IM , Kothari A , Krummenacker M , Midford PE *et al*., The MetaCyc database of metabolic pathways and enzymes – a 2019 update. Nucleic Acids Res 48:D445–D453 (2020). 10.1093/nar/gkz862. 31586394 PMC6943030

[78] Toraya T , Yongsmith B , Tanaka A and Fukui S , Vitamin B12 production by a methanol‐utilizing bacterium. Appl Microbiol 30:477 (1975). 10.1128/am.30.3.477-479.1975. 1180552 PMC187207

[79] Lukáčová A , Beck T , Koptašiková L , Benda A , Tomečková L , Trniková M *et al*., Euglena gracilis can grow in the mixed culture containing Cladosporium westerdijkiae, Lysinibacillus boronitolerans and Pseudobacillus badius without the addition of vitamins B1 and B12. J Biotechnol 351:50–59 (2022). 10.1016/j.jbiotec.2022.04.013. 35500702

[80] Rodionov DA , Arzamasov AA , Khoroshkin MS , Iablokov SN , Leyn SA , Peterson SN *et al*., Micronutrient requirements and sharing capabilities of the human gut microbiome. Front Microbiol 10:1316 (2019). 10.3389/fmicb.2019.01316. 31275260 PMC6593275

[81] Hosmani SS , Singh D , Rathod V , Ravi M , Rayudu K , Narmada S *et al*., Keratinase production by Endophytic bacteria Aneurinibacillus aneurinilyticus VRCS‐4 isolated from xerophytic plant Opuntia ficus‐indica (prickly pear). South Asian J Exp Biol 11:725–732 (2021). 10.38150/sajeb.11(6).p725-732.

[82] Syed B , Nagendra Prasad MN and Satish S , Synthesis and characterization of silver nanobactericides produced by Aneurinibacillus migulanus 141, a novel endophyte inhabiting Mimosa pudica L. Arab J Chem 12:3743–3752 (2019). 10.1016/j.arabjc.2016.01.005.

[83] Patel S and Gupta RS , A phylogenomic and comparative genomic framework for resolving the polyphyly of the genus bacillus: proposal for six new genera of Bacillus species, Peribacillus gen. nov., Cytobacillus gen. nov., Mesobacillus gen. nov., Neobacillus gen. nov., Metabacillus gen. nov. and Alkalihalobacillus gen. nov. Int J Syst Evol Microbiol 70:406–438 (2020). 10.1099/ijsem.0.003775. 31617837

[84] Gupta RS , Patel S , Saini N and Chen S , Robust demarcation of 17 distinct Bacillus species clades, proposed as novel Bacillaceae genera, by phylogenomics and comparative genomic analyses: description of Robertmurraya kyonggiensis sp. nov. and proposal for an emended genus bacillus limiting it only to the members of the subtilis and cereus clades of species. Int J Syst Evol Microbiol 70:5753–5798 (2020). 10.1099/ijsem.0.004475. 33112222

[85] Wolf JB and Brey RN , Isolation and genetic characterizations of bacillus megaterium cobalamin biosynthesis‐deficient mutants. J Bacteriol 166:51–58 (1986). 10.1128/jb.166.1.51-58.1986. 3082859 PMC214555

[86] Raux E , Lanois A , Warren MJ , Rambach A and Thermes C , Cobalamin (vitamin B12) biosynthesis: identification and characterization of a bacillus megaterium cobI operon. Biochem J 335:159–166 (1998). 10.1042/bj3350159. 9742225 PMC1219764

[87] Mohammed Y , Lee B , Kang Z and Du G , Development of a two‐step cultivation strategy for the production of vitamin B12 by bacillus megaterium. Microb Cell Fact 13:1–10 (2014). 10.1186/s12934-014-0102-7. 25023574 PMC4105875

[88] Wu Q , Zhu L , Jiang L , Xu X , Xu Q , Zhang Z *et al*., Draft genome sequence of Paenibacillus dauci sp. nov., a carrot‐associated endophytic actinobacteria. Genomics Data 5:241–253 (2015). 10.1016/j.gdata.2015.06.010. 26484263 PMC4583675

[89] Zhu L , Wu Q , Xu Q , Xu X , Jiang L and Huang H , Draft genome sequence of Paenibacillus algorifonticola sp. nov., an antimicrobial‐producing strain. Genomics Data 5:302–308 (2015). 10.1016/j.gdata.2015.06.023. 26484273 PMC4583682

[90] Chhe C , Uke A , Baramee S , Tachaapaikoon C , Pason P , Waeonukul R *et al*., Characterization of a thermophilic facultatively anaerobic bacterium Paenibacillus sp. strain DA‐C8 that exhibits xylan degradation under anaerobic conditions. J Biotechnol 342:64–71 (2021). 10.1016/j.jbiotec.2021.10.008. 34688788

[91] Xia W , Chen W , Peng W f and Li K t , Industrial vitamin B12 production by Pseudomonas denitrificans using maltose syrup and corn steep liquor as the cost‐effective fermentation substrates. Bioprocess Biosyst Eng 38:1065–1073 (2015). 10.1007/s00449-014-1348-5. 25561346

[92] Arasu MV , Sarkar R , Sekar BS , Kumar V , Rathnasingh C , Choi JDR *et al*., Isolation of a novel pseudomonas species SP2 producing vitamin B 12 under aerobic condition. Biotechnol Bioprocess Eng 18:43–51 (2013). 10.1007/s12257-012-0518-z.

[93] Martens JH , Barg H , Warren M and Jahn D , Microbial production of vitamin B12. Appl Microbiol Biotechnol 58:275–285 (2002). 10.1007/s00253-001-0902-7. 11935176

[94] Li KT , Liu DH , Chu J , Wang YH , Zhuang YP and Zhang SL , An effective and simplified pH‐stat control strategy for the industrial fermentation of vitamin B12 by Pseudomonas denitrificans. Bioprocess Biosyst Eng 31:605–610 (2008). 10.1007/s00449-008-0209-5. 18320234

[95] Peng W , Cheng X , Zhang H y and Li KT , the metabolic characteristicsof high‐production vitamin B12 by Pseudomonas denitrificans under dissolved oxygen step‐wise reduction. J Chem Technol Biotechnol 89:1396–1401 (2014). 10.1002/jctb.4219.

[96] Sych JM , Lacroix C and Stevens MJA , Vitamin B12– physiology, production and application. Ind Biotechnol Vitam, Biopigm, Antioxid 129–159 (2016). 10.1002/9783527681754.ch6.

[97] Maher JM , Markey JC and Ebert‐May D , The other half of the story: effect size analysis in quantitative research. CBE Life Sci Educ 12:345–351 (2013). 10.1187/cbe.13-04-0082. 24006382 PMC3763001

[98] Patz S , Rauh M , Gautam A and Huson DH , mgPGPT: Metagenomic analysis of plant growth‐promoting traits. bioRxiv 2024 (2024). 10.1101/2024.02.17.580828.

[99] Khare E , Mishra J and Arora NK , Multifaceted interactions between endophytes and plant: developments and prospects. Front Microbiol 9:2732 (2018). 10.3389/fmicb.2018.02732. 30498482 PMC6249440

[100] Compant S , Saikkonen K , Mitter B , Campisano A and Mercado‐Blanco J , Editorial special issue: soil, plants and endophytes. Plant Soil 405:1–11 (2016). 10.1007/s11104-016-2927-9.

