# Supplementary material for: Lettuce fortification through vitamin B12 ‐producing bacteria – proof of concept study
Source: J Sci Food Agric. 2025 Jan 20;105(6):3343–54. doi: 10.1002/jsfa.14095 (PMC11949862; doi:10.1002/jsfa.14095)
Supplement: Supplementary file 1 — Fig. S1. Structure of cobalamin. Ra = deoxyadenosine which forms adenosylcobalamin (AdoCbl); Rb = cyano group which forms cyanocobalamin (CNCbl); Rc = deoxyadenosine which forms methylcobalamin (MeCbl), Rd = hoxidrile group which forms hydroxycobalamin (OHCbl). Designed with www.reaxys.com. Fig. S2. Krona plot representation of the major PGPTs found in Methylobacterium sp. P1‐11, generated with PLaBase web tool. Fig. S3. IGS‐RFLP patterns (agarose gel 2.5%) obtained from Methylobacterium sp. P1‐11 and from bacteria isolated from lettuce (Let1‐Let10) treated with this strain. The patterns were obtained using AluI (left)and HhaI (right) endonucleases. [file JSFA-105-3343-s001.docx]

*
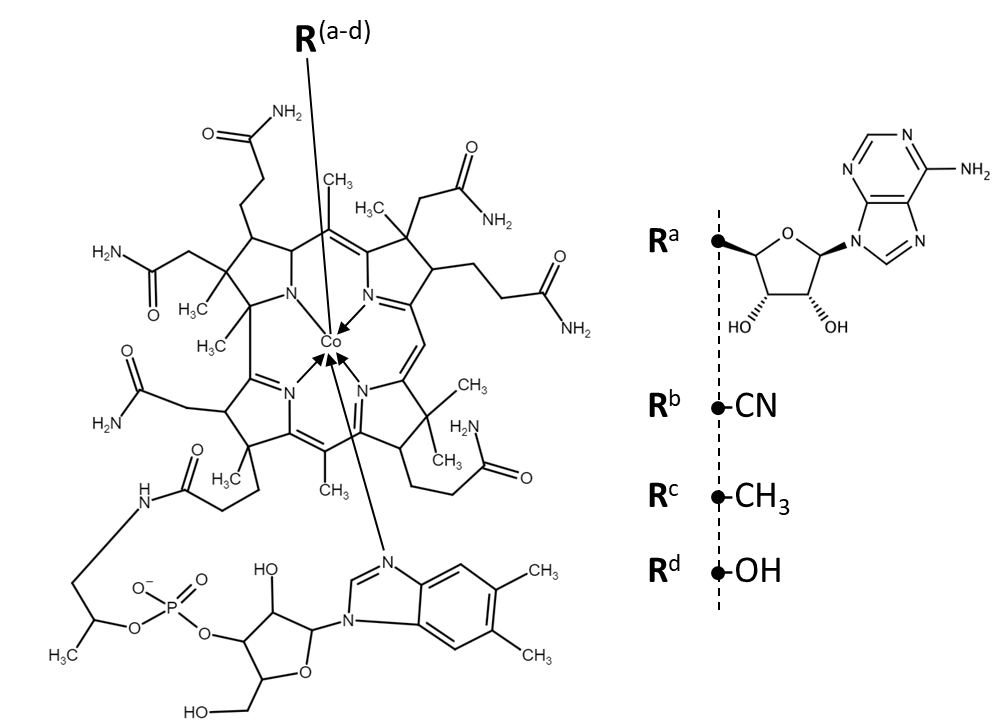
*

**Figure S1**: Structure of cobalamin. R^a^ = deoxyadenosine which forms adenosylcobalamin (AdoCbl); R^b^ = cyano group which forms cyanocobalamin (CNCbl); R^c^ = deoxyadenosine which forms methylcobalamin (MeCbl), R^d^ = hoxidrile group which forms hydroxycobalamin (OHCbl). Designed with [www.reaxys.com](http://www.reaxys.com).


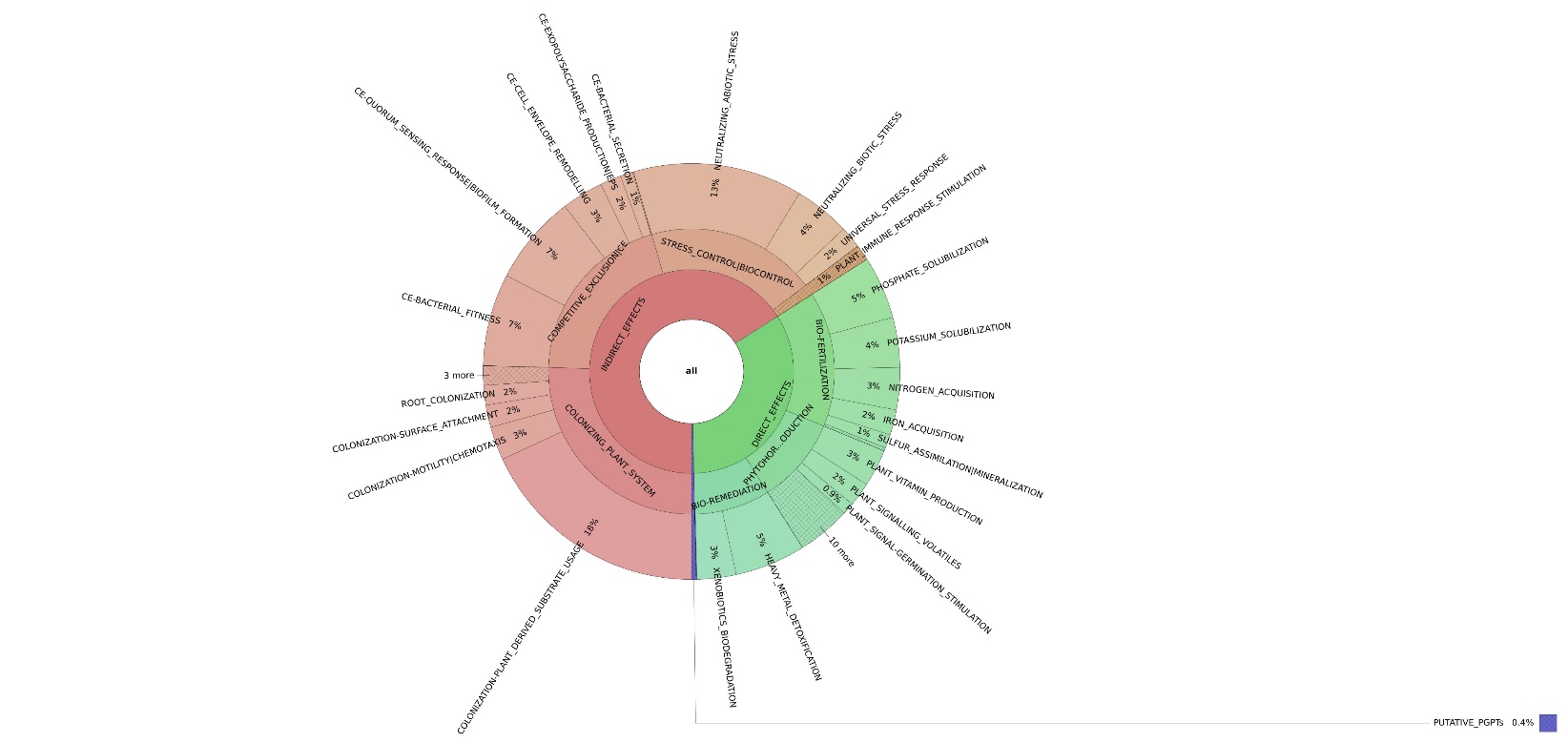


**Figure S2:** Krona plot representation of the major PGPTs found in Methylobacterium sp. P1-11, generated with PLaBase web tool.


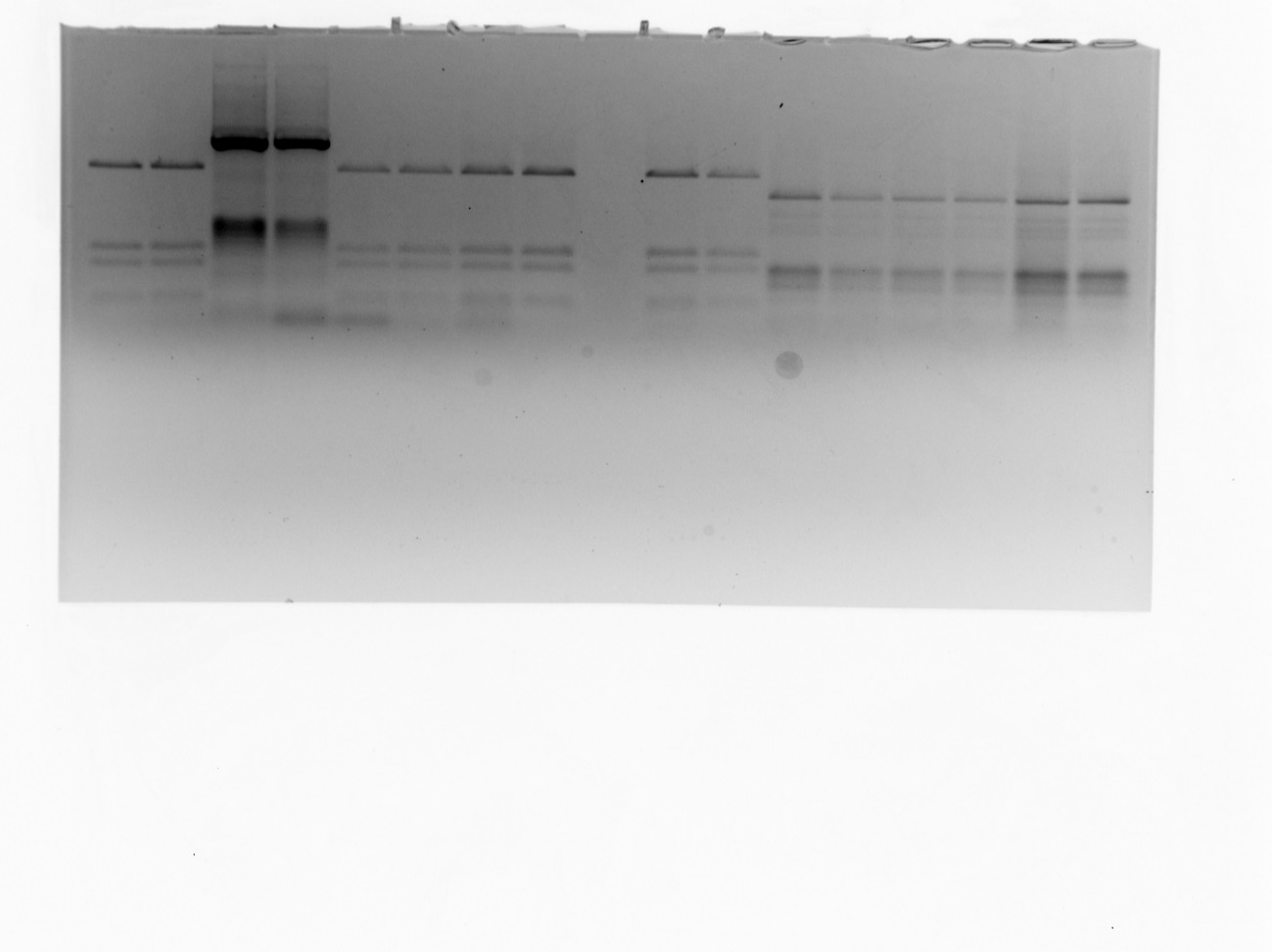

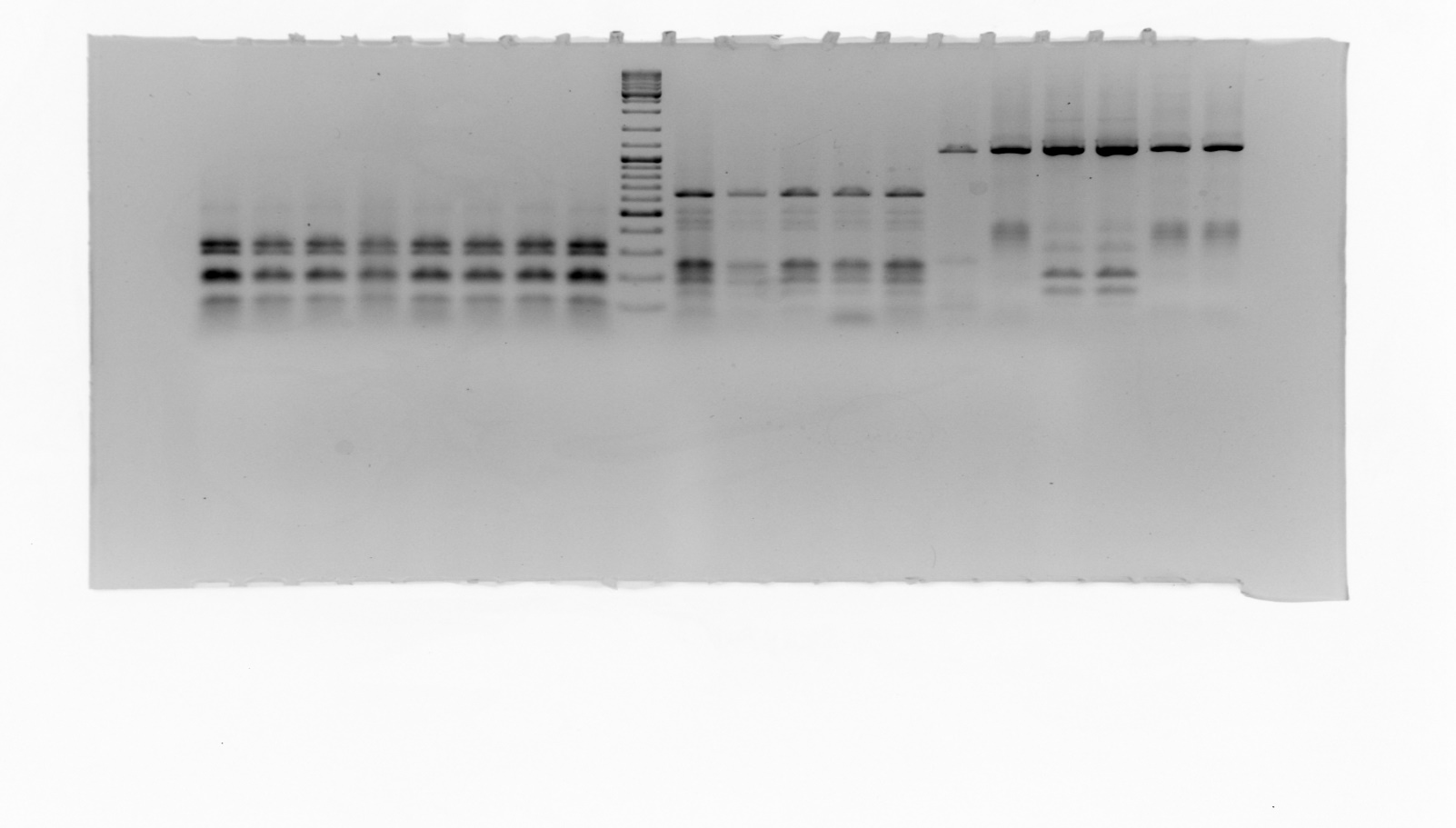


P1-11

Let1

Let2

Let4

Let3

Let5

Let6

Let7

Let9

Let8

Let10

*Hha-I*


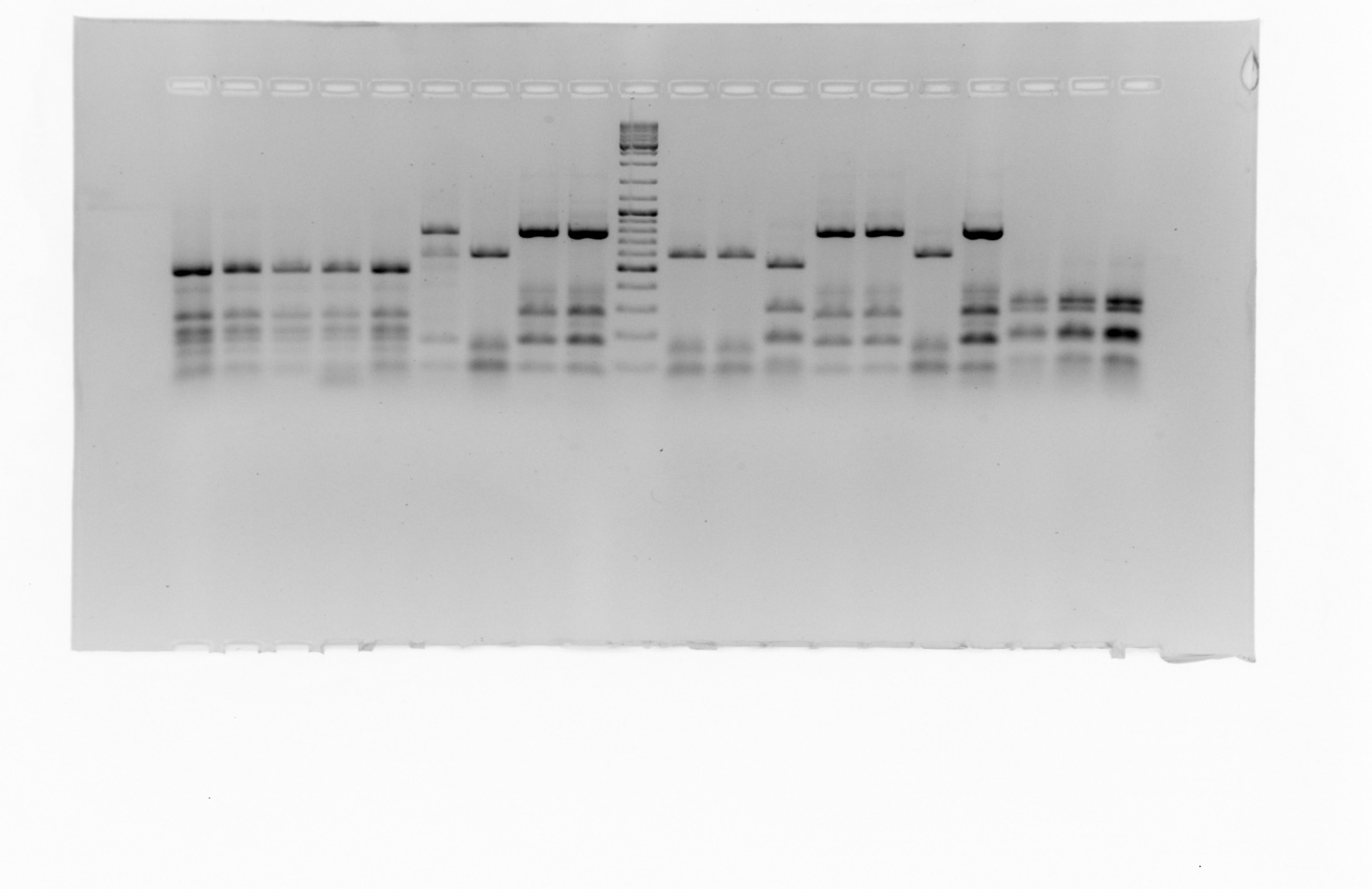

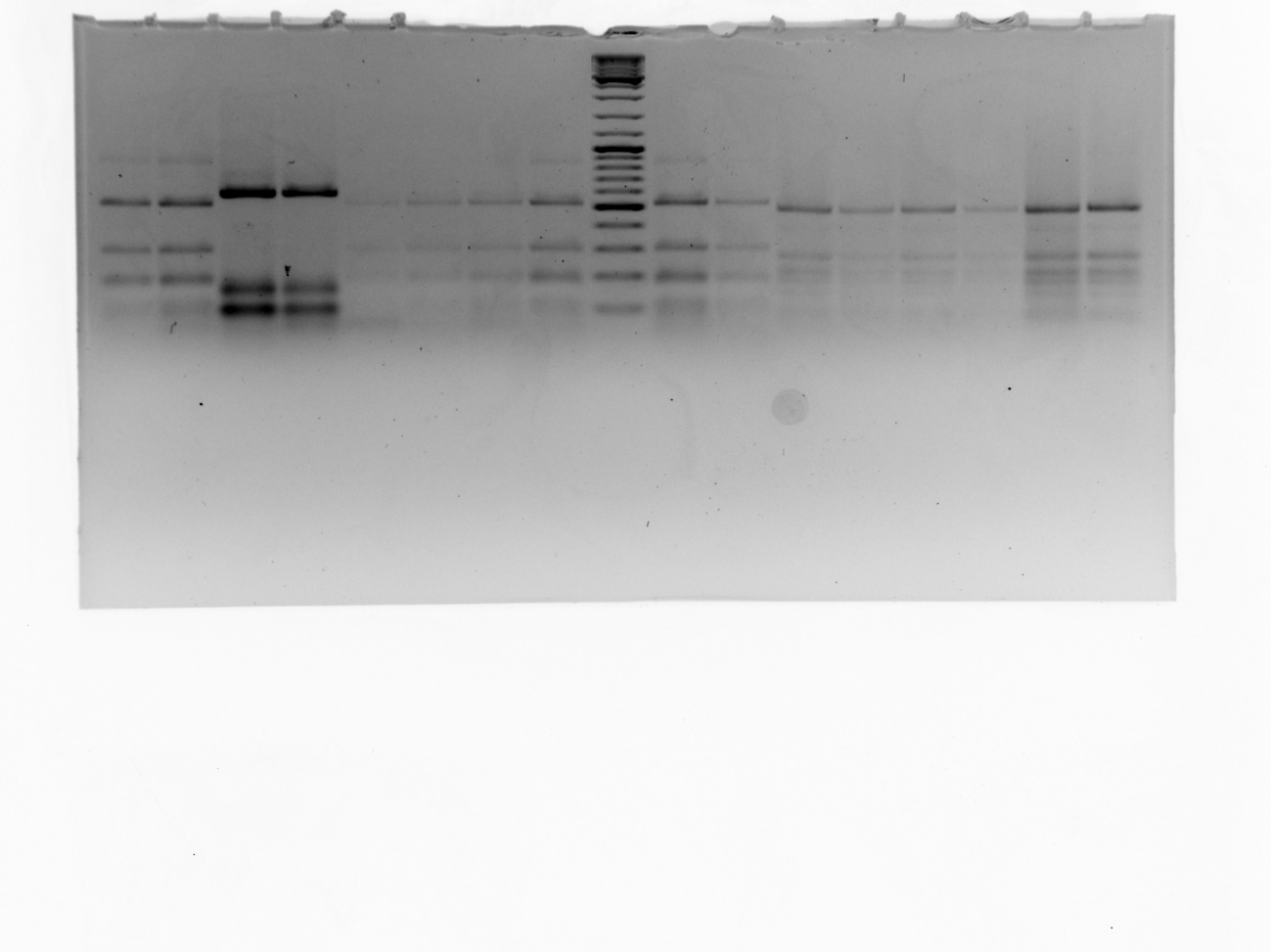


*Alu-I*

P1-11

Let1

Let2

Let4

Let3

Let5

Let6

Let7

Let9

Let8

Let10

**Figure S3:** IGS-RFLP patterns (agarose gel 2.5%) obtained from Methylobacterium sp. P1-11 and from bacteria isolated from lettuce (Let1-Let10) treated with this strain. The patterns were obtained using AluI (left)and HhaI (right) endonucleases.
