# Supplementary material for: Lettuce fortification through vitamin B12 ‐producing bacteria – proof of concept study
Source: J Sci Food Agric. 2025 Jan 20;105(6):3343–54. doi: 10.1002/jsfa.14095 (PMC11949862; doi:10.1002/jsfa.14095)
Supplement: Supplementary file 2 — Table S1. All the 65 bacterial endophytic strains from the Bioresources strain collection of the AIT Austrian Institute of Technology, selected for this study. For each strain are reported, the AIT strain collection ID, the scientific name, the strain name, the matrix from where it was isolated, the genome length, its completeness, and the percentage of GC. [file JSFA-105-3343-s002.docx]

**Table S1:** All the 65 bacterial endophytic strains from the Bioresources strain collection of the AIT Austrian Institute of Technology, selected for this study. For each strain are reported, the AIT strain collection ID, the scientific name, the strain name, the matrix from where it was isolated, the genome length, its completeness, and the percentage of GC.

| AIT Strain Collection | Taxonomic characterization | | Strain name | | | Source | | | Genome lenght (bp) | | | Complet  eness | | | GC% | | |  |
| --- | --- | --- | --- | --- | --- | --- | --- | --- | --- | --- | --- | --- | --- | --- | --- | --- | --- | --- |
| Ref. strain | *Pseudomonas denitrificans* | | ATCC 13867 | | | Unknown | | | 5696307 | | | *100* | | | 65.24 | | |  |
| *-* | *Bacillus altitudinis* | | EKB069BA17 | | | Unknown | | | 3781441 | | | *99.59* | | | 41.3 | | |  |
| *-* | *Bacillus subtilis* | | EKB044BA17 | | | Unknown | | | 4282901 | | | *99.81* | | | 43.4 | | |  |
| *-* | *Pantoea agglomerans* | | LLC 4-5 | | | Unknown | | | 4730913 | | | *100* | | | 55.3 | | |  |
| 1133 | *Paenibacillus polymyxa* | | CCI-25 | | | Compost | | | 5605774 | | | *99.85* | | | 45.61 | | |  |
| 1135 | *Bacillus atrophaeus* | | 176s | | | *Tortella tortuosa* | | | 4292434 | | | *99.17* | | | 43.13 | | |  |
| 1142 | *Bacillus pumilus* | | P1-01 | | | *Glycine max* | | | 3785273 | | | *99.59* | | | 41.4 | | |  |
| 1173 | *Pseudomonas atacamensis* | | H17_3 | | | *Solanum tuberosum* | | | 6028305 | | | *99.66* | | | 60 | | |  |
| 1176 | *Pseudomonas oryzihabitans* | | H17_3 | | | *Solanum tuberosum* | | | 5049236 | | | *99.67* | | | 65.9 | | |  |
| 1183 | *Bacillus pumilus* | | P2-05 | | | *Glycine max* | | | 3749221 | | | *99.59* | | | 41.5 | | |  |
| 1265 | *Bacillus pumilus* | | E3-05 | | | *Glycine max* | | | 3833889 | | | *99.48* | | | 41.3 | | |  |
| 1353 | *Bacillus subtilis* | | S1-05 | | | *Glycine max* | | | 4012109 | | | *99.81* | | | 43.7 | | |  |
| 1370 | *Paenibacillus polymyxa* | | S3-27 | | | *Glycine max* | | | 5675599 | | | *99.85* | | | 45.7 | | |  |
| 1390 | *Priestia megaterium* | | M3-23 | | | *Glycine max* | | | 6073146 | | | *99.43* | | | 37.3 | | |  |
| 1412 | *Bacillus pumilus* | | BV-A-43-12 | | | *Beta vulgaris* | | | 3696628 | | | *99.59* | | | 41.3 | | |  |
| 1417 | *Bacillus altitudinis* | | EKA006BA16 | | | Earthworm soil | | | 3804684 | | | *99.59* | | | 41.2 | | |  |
| 1419 | *Bacillus subtilis* | | EKA023BA16 | | | Earthworm soil | | | 4151560 | | | *99.81* | | | 43.4 | | |  |
| 1420 | *Lysinibacillus fusiformis* | | EKA045BA16 | | | Earthworm soil | | | 4543853 | | | *99.34* | | | 37.6 | | |  |
| 1421 | *Paenibacillus polymyxa* | | EKA078BA16 | | | Earthworm soil | | | 5656086 | | | *99.85* | | | 45.4 | | |  |
| 1425 | *Bacillus subtilis* | | EKB010BA16 | | | Compost | | | 4162665 | | | *99.81* | | | 43.6 | | |  |
| 1426 | *Bacillus subtilis* | | EKB032BA16 | | | Compost | | | 4323077 | | | *99.81* | | | 43.3 | | |  |
| 1427 | *Bacillus licheniformis* | | EKB057BA16 | | | Compost | | | 4177461 | | | *98.96* | | | 46.2 | | |  |
| 1435 | *Bacillus altitudinis* | | EKB151BA16 | | Compost | | | 3756234 | | | *99.59* | | | 41.2 | | |  |  |
| 1452 | | *Pseudomonas orientalis* | | LC41_6 | | | *Solanum tuberosum* | | | 6605808 | | | *99.8* | | | 59.2 | | |
| 1489 | | *Pseudomonas sp.* | | F1_10 | | | *Solanum tuberosum* | | | 5800186 | | | *99.93* | | | 60.5 | | |
| 1492 | | *Pseudomonas crudilactis* | | F1_13 | | | *Solanum tuberosum* | | | 6747472 | | | *99.8* | | | 59.1 | | |
| 1548 | | *Pseudomonas fluorescens* | | F17_14 | | | *Solanum tuberosum* | | | 6781446 | | | *99.93* | | | 60.5 | | |
| 1925 | | *Priestia megaterium* | | A3-11 | | | *Glycine max* | | | 7263097 | | | *99.43* | | | 37.1 | | |
| 1940 | | *Methylobacterium sp.* | | P1-11 | | | *Solanum tuberosum* | | | 6774627 | | | *100* | | | 69 | | |
| 1994 | | *Tardiphaga sp.* | | P9-11 | | | *Solanum tuberosum* | | | 6056105 | | | *99.6* | | | 61.3 | | |
| 2034 | | *Pseudomonas sp.* | | H17_3 | | | *Solanum tuberosum* | | | 6588949 | | | *99.93* | | | 59.3 | | |
| 2070 | | *Pseudomonas kilonensis* | | Eca B17 | | | *Solanum tuberosum* | | | 6733336 | | | *100* | | | 60.6 | | |
| 2136 | | *Paenibacillus amylolyticus* | | LZA 5-1 | | | *Triticum aestivum* | | | 7259596 | | | *99.85* | | | 45.4 | | |
| 2136 | | *Pantoea agglomerans* | | LZA 5-1 | | | *Triticum aestivum* | | | 4730791 | | | *100* | | | 55.3 | | |
| 2141 | | *Kocuria rhizophila* | | LLC 5-4 | | | *Triticum aestivum* | | | 2727559 | | | *99.34* | | | 70.6 | | |
| 2143 | | *Metabacillus dongyingensis* | | KLA 4-2 | | | *Triticum aestivum* | | | 5764774 | | | *99.56* | | | 39.5 | | |
| 2145 | | *Paenibacillus xylanexedens* | | KLA 4-4 | | | *Triticum aestivum* | | | 7296266 | | | *99.71* | | | 45.6 | | |
| 2146 | | *Bacillus velezensis* | | KLA 5-1 | | | *Triticum aestivum* | | | 3998529 | | | *99.8* | | | 46.3 | | |
| 2150 | | *Paenibacillus amylolyticus* | | KLC 4-2 | | | *Triticum aestivum* | | | 6976237 | | | *99.85* | | | 45.8 | | |
| 2154 | | *Paenibacillus sp.* | | KLC 4-12 | | | *Triticum aestivum* | | | 6431219 | | | *99.85* | | | 44.6 | | |
| 2155 | | *Paenibacillus sp.* | | KLC 4-13 | | | *Triticum aestivum* | | | 7077434 | | | *99.85* | | | 45.8 | | |
| 2161 | | *Paenibacillus sp.* | | KLC 5-3 | | | *Triticum aestivum* | | | 7077396 | | | *99.85* | | | 45.8 | | |
| 2165 | | *Bacillus velezensis* | | LZC 5-1 | | | *Triticum aestivum* | | | 4073970 | | | *99.81* | | | 45.9 | | |
| 2169 | | *Paenibacillus nuruki* | | KZC 4-1 | | | *Triticum aestivum* | | | 5103052 | | | *99.45* | | | 39.3 | | |
| 2170 | | *Pantoea agglomerans* | | KZC 4-2 | | | *Triticum aestivum* | | | 4954203 | | | *100* | | | 54.8 | | |
| 2171 | | *Pseudomonas poae* | | KZC 4-3 | | | *Triticum aestivum* | | | 5726537 | | | *99.86* | | | 60.7 | | |
| 2175 | | *Pantoea agglomerans* | | KZC 5-3 | | | *Triticum aestivum* | | | 4953509 | | | *100* | | | 54.8 | | |
| 2969a | | *Bacillus safensis* | | M3-22a | | | *Glycine max* | | | 3832250 | | | *99.59* | | | 41.5 | | |
| 2969b | | *Priestia megaterium* | | M3-22b | | | *Glycine max* | | | 6070227 | | | *100* | | | 37.4 | | |
| 4237 | | *Peribacillus frigoritolerans* | | G65BA17 | | | Unknown | | | 5773514 | | | *98.91* | | | 40.3 | | |
| 4238 | | *Peribacillus frigoritolerans* | | G66BA17 | | | Unknown | | | 5794529 | | | *98.91* | | | 40 | | |
| 4242 | | *Bacillus halotolerans* | | S11BA17 | | | Unknown | | | 4253821 | | | *99.29* | | | 43.5 | | |
| 4245 | | *Bacillus subtilis* | | S18BA17 | | | Unknown | | | 4254201 | | | *99.81* | | | 43.2 | | |
| 4249 | | *Pseudomonas fluorescens* | | S26BA17 | | | Unknown | | | 3773281 | | | *99.07* | | | 46.3 | | |
| 4251 | | *Bacillus siamensis* | | S23BA17 | | | Unknown | | | 6868092 | | | *99.8* | | | 60.6 | | |
| 4252 | | *Bacillus subtilis* | | S24BA17 | | | Unknown | | | 4253388 | | | *99.81* | | | 43.2 | | |
| 4261 | | *Aneurinibacillus migulanus* | | C8BA17 | | | Unknown | | | 6059939 | | | *99.22* | | | 43.2 | | |
| 4262 | | *Bacillus halotolerans* | | C9BA17 | | | Unknown | | | 4251341 | | | *99.29* | | | 43.5 | | |
| 4264 | | *Bacillus licheniformis* | | C11BA17 | | | Unknown | | | 4266396 | | | *98.86* | | | 45.9 | | |
| 4265 | | *Lysinibacillus fusiformis* | | C13BA17 | | | Unknown | | | 4869393 | | | *99.34* | | | 37.1 | | |
| 4266 | | *Bacillus halotolerans* | | C15BA17 | | | Unknown | | | 4157630 | | | *99.66* | | | 43.6 | | |
| 4277 | | *Paenibacillus sp.* | | O21BA17 | | | Unknown | | | 6870735 | | | *99.85* | | | 46.1 | | |
| 4280 | | *Paenibacillus sp.* | | O41BA17 | | | Unknown | | | 6833276 | | | *99.85* | | | 46.1 | | |
| 4283 | | *Bacillus halotolerans* | | O61BA17 | | | Unknown | | | 4252502 | | | *99.29* | | | 43.5 | | |
| 4284 | | *Bacillus halotolerans* | | G4BA17 | | | Unknown | | | 4252322 | | | *99.29* | | | 43.5 | | |
| 4289 | | *Priestia aryabhattai* | | A3-14 | | | Unknown | | | 5287971 | | | *99.43* | | | 37.9 | | |
